# Additive SMILES-Based Carcinogenicity Models: Probabilistic Principles in the Search for Robust Predictions

**DOI:** 10.3390/ijms10073106

**Published:** 2009-07-08

**Authors:** Andrey A. Toropov, Alla P. Toropova, Emilio Benfenati

**Affiliations:** 1 Institute of Geology and Geophysics, 100041, Khodzhibaev St. 49, Tashkent, Uzbekistan; E-Mail: altoropova@mail.ru (A.P.T.); 2 Istituto di Ricerche Farmacologiche Mario Negri, 20156, Via La Masa 19, Milano, Italy; E-Mail: benfenati@marionegri.it (E.B.)

**Keywords:** QSAR, SMILES, optimal descriptor, carcinogenicity, balance of correlations, applicability domain

## Abstract

Optimal descriptors calculated with the simplified molecular input line entry system (SMILES) have been utilized in modeling of carcinogenicity as continuous values (logTD_50_). These descriptors can be calculated using correlation weights of SMILES attributes calculated by the Monte Carlo method. A considerable subset of these attributes includes rare attributes. The use of these rare attributes can lead to overtraining. One can avoid the influence of the rare attributes if their correlation weights are fixed to zero. A function, limS, has been defined to identify rare attributes. The limS defines the minimum number of occurrences in the set of structures of the training (subtraining) set, to accept attributes as usable. If an attribute is present less than limS, it is considered “rare”, and thus not used. Two systems of building up models were examined: 1. classic training-test system; 2. balance of correlations for the subtraining and calibration sets (together, they are the original training set: the function of the calibration set is imitation of a preliminary test set). Three random splits into subtraining, calibration, and test sets were analysed. Comparison of abovementioned systems has shown that balance of correlations gives more robust prediction of the carcinogenicity for all three splits (split 1: r_test_^2^=0.7514, s_test_=0.684; split 2: r_test_^2^=0.7998, s_test_=0.600; split 3: r_test_^2^=0.7192, s_test_=0.728).

## 1. Introduction

Carcinogenicity is an important endpoint from a toxicological point of view and quantitative structure – activity relationships (QSAR) are a tool for modeling this endpoint [1–3]. Usually, the QSAR analysis is based on molecular descriptors, calculated from molecular graphs [3,4]. However, the simplified molecular input line entry system (SMILES) [5–7] has become a prospective alternative to molecular graphs in QSAR analysis [8–11], owing to an expansion of the databases available via the Internet with molecular structures given in SMILES notation [15,16]. The present study aimed to estimate the ability of the SMILES-based optimal descriptors to be a tool for QSAR analysis of carcinogenicity of non-congeneric chemicals.

## 2. Materials and Methods

*Carcinogenicity data*: Experimental values for carcinogenicity were taken from publicly available data sources and further checked for chemical structures [17]. Carcinogenicity is expressed as the potency dose that induces cancer in rats (TD_50_, in mg/kg body weight). These values have been converted into mmol/kg body weight. The -log(TD_50_) was examined as endpoint for the modelling. Initially, 401 chemicals have been extracted from [17]. These compounds were selected as substances with numerical data on the carcinogenicity available from [17].

However, this set (401 compounds) contains eight outliers (Table 1): for these compounds the difference between experimental and calculated (by our approach) value of -logTD50 is more than the double the standard error (2s). Probably the high symmetry and the presence of the *N*-nitroso group can lead to the unusual behaviour of these substances. These compounds were removed. Thus, 393 compounds were examined in this study. SMILES notations which were used in this study have been taken from [18].

We randomly split these 393 chemicals three times into training (n=165), calibration (n=167) and test (n=61) sets. The range of -log(TD_50_) values for these sets is about from −2 to 5 logarithmic units. Below, these splits are denoted the Split1, Split2, and Split3 (The *Supplementary Materials* contain lists of these splits).

The modification of the descriptor that was used for modeling bee toxicity [10] is the tool for QSAR analysis of the carcinogenicity. This descriptor is calculated as follows:

(1) $$\text{DCW}(\text{lim}\text{S})=\text{CW}(\text{dC})+\Sigma \;\text{CW}{(}^{1}{\text{SA}}_{\text{k}})+\Sigma \;\text{CW}{(}^{2}{\text{SA}}_{\text{k}})+\Sigma \;\text{CW}{(}^{3}{\text{SA}}_{\text{k}})$$

where ^1^SA_k_, ^2^SA_k_, ^3^SA_k_ are SMILES attributes. ^1^SA_k_, ^2^SA_k_, and ^3^SA contain one, two, and three SMILES elements, respectively. The SMILES element can be one (e.g., ‘C’, ‘c’, ‘N’, ‘S’, etc.), two (e.g., ‘Cl’, ‘Br’, etc.), three (‘C=O’), and four symbols (‘[O−]’). The order of elements in depiction of the ^2^SA_k_ or ^3^SA_k_ is defined by the ASCII characters. In other words only one version of AB-sequence or ABC-sequence is possible in the list of the SMILES-attributes (not AB together with BA, or ABC together with CBA).

The dC is the difference of the number of ‘C’ (capital letter) in the given SMILES notation minus the number of ‘c’ (lowercase letter) in the given SMILES notation. For example, this global SMILES attribute is denoted as ‘!001’, if dC=N(‘c’) – N(‘C’)=1, and as ‘!-02’ if the dC =−2. The CW(dC) is the correlation weight of the dC. The symbol “C” (capital letter) is the representation of a carbon atom in the sp^3^ configuration. The symbol “c” (lowercase letter) is the representation of a carbon atom in sp^2^ configuration. Thus, the dC is a measure of presence of rigid and flexible fragments in molecular architecture. The examined substances contain chlorine that gives an additional ‘C’. The chlorine is not rigid fragment in molecular system and we have calculated the dC taking into account the ‘C’ from chlorine atoms. Table 2 contains an example of the representation of SMILES by the set of SMILES attributes.

The CW(dC), CW(^1^SA_k_), CW(2SA_k_), and CW(3SA_k_) are correlation weights of the above SMILES attributes. By means of the Monte Carlo method one can calculate numerical data for these weights which give maximal value of determination coefficient (square of the correlation coefficient, r^2^) for the training set. However, most probably overtraining will result, i.e., an excellent model on the training set will be accompanied by a poor model for the test set. In order to avoid overtraining one can use the correlation balance [11], i.e., split the available chemicals into three sets: subtraining, calibration, and external test set. This approach gave reasonable result for the case of toxicity of 61 compounds [11], however for carcinogenicity of 393 compounds it is not enough. The use of the correlation balance and blocking of rare SMILES attributes [10] can improve the model. The blocking of rare attributes can be done by the scheme: if the number of SMILES from the training (subtraining) set which contain the SMILES attribute SA* is less than the limS, the correlation weight of the SA* should be fixed equal to zero, CW(SA*)=0.

**Figure 1.**
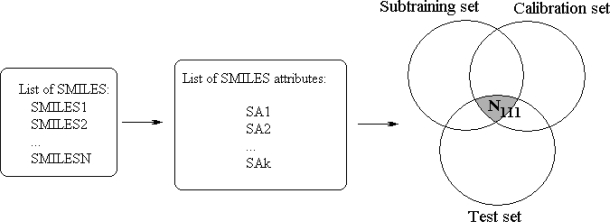

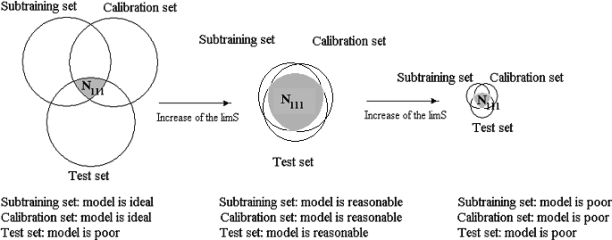
General scheme of construction of the optimal SMILES-based descriptors by means of the correlation balance method. **Phase 1.** The definition of general list of the SMILES attributes (limS=0). The N_111_ is the number of the attributes which are present in the subtraining, in calibration, and in test set. If limS=0 the N_111_ is relatively low. **Phase 2.** The definition of the most productive limS value: 0 < limS* < ∞; this value gives maximum of the N_111_, i.e., number of the SMILES attributes which are present in the subtraining, in calibration, and in test set.

Without rare attributes the model becomes better for the external test set. However, if limS is too large, the predictive potential of the model decreases, because the low number of active SMILES attribute cannot provide a high quality model. Thus, the central point of the system of modeling is the selection of the most efficient limS. The general scheme of the construction of optimal SMILES-based descriptors by the correlation balance method is represented in Figure 1.

This system can be denoted as a **[Subtraining-Calibration-Test] system.** The model can be satisfactory if the N_111_, i.e., the number of active (not blocked) attributes which are present in subtraining, calibration, and test sets, is as large as possible. The more traditional, “classic” approach is the construction of the model using united training set to predict the endpoint for an external test set. This system can be denoted as **[Training-Test] system.** This model can be satisfactory if the N_101_, i.e., the number of active attributes which are present in both the training and test set is as large as possible.

The correlation weights were calculated by the Monte Carlo method Optimization. The **[Training-Test] system** is based on correlation weights which provide maximum of the correlation coefficient between DCW(limS) and log(TD_50_) for the training set. The **[Subtraining-Calibration-Test] system** is based on correlation weights which provide the maximum of a target function (TF) calculated as follows [11–14]:

(2) TF=D(subtraining)+D(calibration)−ABS[D(subtraining)+D(calibration)] 0.1

where D(subtraining) and D(calibration) are determination coefficients between DCW(limS) and log(TD_50_) for subtraining and calibration sets, respectively. Thus the optimization for the above system has been carried out by the same algorithm [11], but with different target functions.

For each attribute SA, CW(SA) is determined initially by setting the start values of all CWs to 1 ± 0.01*random. The random is the generator of random value of range (0, 1). The regular order of number of attributes (i.e., 1, 2, 3, 4, 5,…) is replaced by a random sequence (e.g., 3, 1, 5, 2, 4,...). A starting value of target function (TF1) is calculated. In a generated random sequence, each attribute correlation weight CWi was modified with the algorithm:

1. DCWi:=0.5*CWi; Eps:=0.1*DCWi;
2. Calculation of TF1; CWi:=CWi + DCWi;
3. Calculation of TF2, after modify CWi;
4. If TF2 > TF1 then TF1:=TF2; go to 2
5. CWi:=CWi - DCWi;
6. DCWi:= −0.5*DCWi;
7. If absolute value (DCWi) >Eps then go to 2.

**Figure 2.**
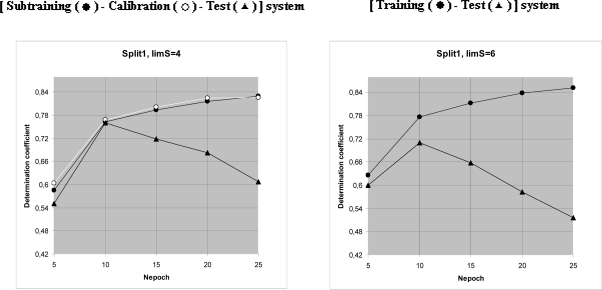

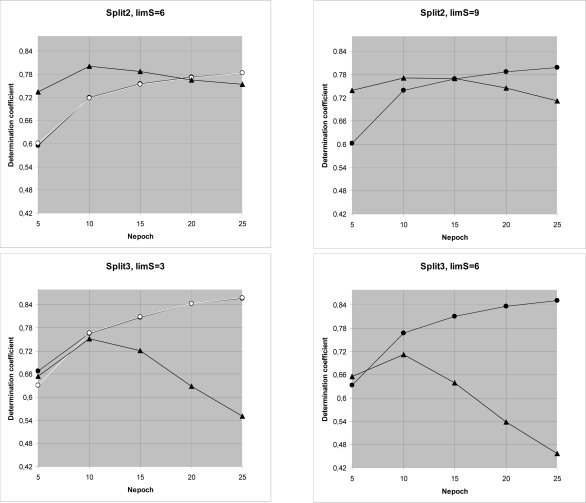
Results of computational experiments, which were used to establish of the preferable number of epochs of the Monte Carlo optimization (Nepoch). Triangles indicate curves for the test sets. Black circles denote the sub training set. White circles denote the calibration set.

Then, steps of 1–7 are carried out for all CWs (the epoch of the optimization). By computational experiment the optimal number of the epochs has been established (Table 3). This number is 10 (Figure 2).

## 3. Results

**Figure 3.**
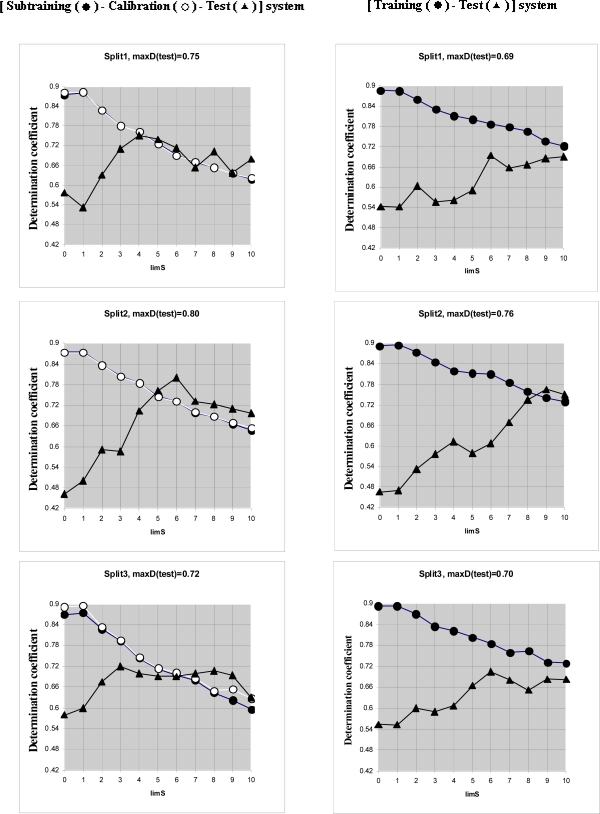
Comparison of the **[subtraining-calibration-test] system** and the **[training-test] system** for three splits.

Computational experiments (Figure 3, Table 4) have shown that **[Subtraining-Calibration-Test]** system gives preferable results in comparison with the **[Training-Test]** system for all three splits. Thus the correlation balance (i.e., **[Subtraining-Calibration-Test]** system) improves QSAR model of log(TD50). It is the second successful experiment using the correlation balance for the QSAR analyses [11].

**Figure 4.**
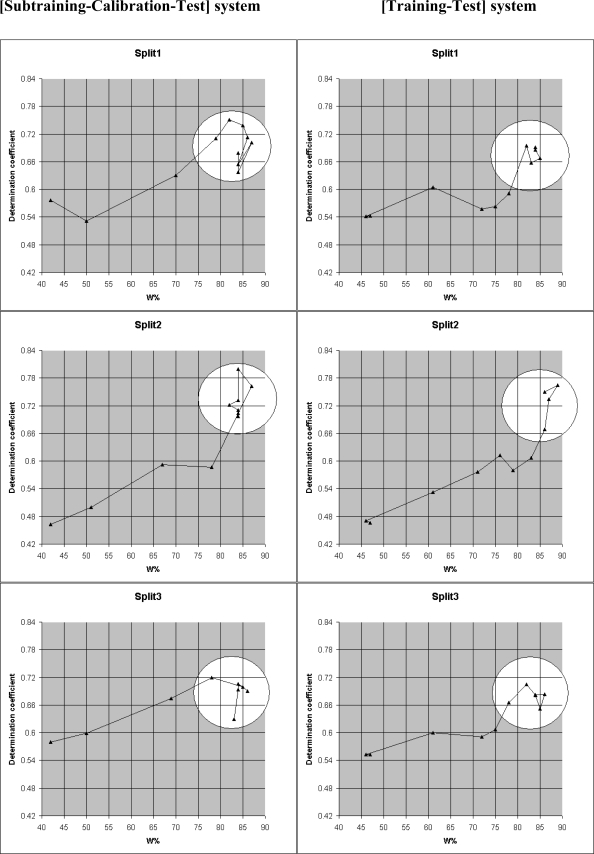
Correlations between the determination coefficient for test set and W% for the three splits (see data from Table 4).

A useful characteristic of these models is W%=N_111_/Nact, where N_111_ is the number of non blocked attributes which take place in subtraining, calibration, and test set; N_act_ is the total number of attributes which are not blocked for a given limS. There is a correlation between W% and the determination coefficient for the test set (Figure 4, Table 4). One can see from the results that good prediction ocurrs if the W% is higher than 80 (excepting **[Subtraining-Calibration-Test ]** for the Split3: in this case W%=78).

The model obtained in the first probe of the Monte Carlo optimization for the split1 with limS=4 is calculated as follows:

(3) $$-\text{log}({\text{TD}}_{50})=-0.5981\;(0.0074)+0.1118\;(0.0004)*\text{DCW}(4)$$

n=165, r^2^=0.7622, s=0.685, F=522 (subtraining set)
n=167, r^2^=0.7620, s=0.734, F=528 (calibration set)
n=61, r^2^=0.7541, s=0.682, F=181 (test set)
Y-scrambling[19,20] for the test set (N_shifting_ =300[20]) gave r^2^_scrambling_ =0.0996

**Figure 5.**
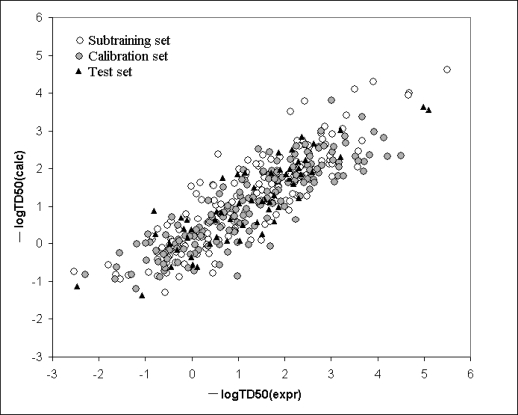
Graphical representation of the model for logTD_50_ calculated with Equation 3.

Figure 5 shows the model calculated with Equation 3, graphically. The *Supplementary Materials* contains numerical data on the experimental and calculated values with Equation 3 (split1 with limS=4). Table 5 contains numerical data on the correlation weights of SMILES attributes obtained in three probes of the Monte Carlo optimization.

## 4. Discussion

One can see that the statistical characteristics of this model are reasonably good. As additional validation we have calculated Y-scrambling criterion, randomly shifting the carcinogenicity values [16,17]. If after the shifting (300 exchanges recommended in Ref.[17]) the correlation coefficient is less than 0.2, the correlation of our model can be classified as not chance correlation. Thus, the Y-scrambling has shown that the Equation 3 gives robust prediction (not chance correlation) for the test set.

In our previous study we examined different equations for the carcinogenicity model, and only one split into the subtraining, calibration and test set [15]. Examination of three splits indicates that good results occur for all three splits (Table 4). Thus, we expect that the present model is more robust, also considering the Y-scrambling test.

One can see from Table 5 that there are three categories of SMILES attributes: category 1 is the set of SMILES attributes with the correlation weight more than zero in all three probes of the Monte Carlo optimization; category 2 is the set of SMILES attributes with the correlation weight less than zero in all three probes; category 3 is the set of SMILES attributes with non consistent values, which have both correlation weights more than zero and correlation weights less zero in the three probes of the optimization. We can say that the category 1 contains promoters of logTD_50_ increase; category 2 contains promoters of logTD_50_ decrease; category 3 contains attributes with unclear influence on logTD_50_.

The !-02, #, Cl, S, [N+], and [O−] SMILES elements are promoters of logTD50 increase, thus of carcinogenicity. However it is necessary to take into account the value of correlation weight as well as the number of the given attribute in the subtraining set. Taking this into account, one can detect that the strongest promoters of the logTD50 increase are Cl (number Cl in the subtraining set is 61, the range of correlation weights of the Cl in three probes is 2.19 – 3.19) and [O−] (the number of [O−] in the subtraining set is 26, the range of correlation weights in three probes is 5.92 – 6.96).

A similar analysis can be done for the promoters of logTD_50_ decrease. For instance, the number of bracket s‘(‘ in the subtraining set is 708 and the range of correlation weights of bracket is from −1.366 till −1.686; the number of ‘=’ in the subtraining set is 77 and the range of correlation weight is from −1.866 till 2.144. Table 6 contains examples of compounds, which contain the mentioned SMILES attributes. Thus, the analysis of the correlation weights of SMILES attributes can help in searching for agents of the carcinogenicity phenomenon.

An important feature of our model is that SMILES attributes are used for the QSAR predicted values and not only as tool for a binary classification (carcinogenic or not). Our model, which provides continuous values, can be used for risk assessment calculations, where a dose is necessary.

The applicability domain for these models can be defined from a probabilistic point of view: one can estimate the carcinogenic potential of compound if the SMILES of this compound does not contain rare SMILES attributes. A stronger definition of the applicability domain can be formulated taking into account the roles of the attributes (as promoters of logTD_50_ increase/decrease): thus, one can estimate the carcinogenic potential of a compound if the SMILES of the compound contains solely apparent promoters of logTD_50_ increase and/or decrease (without of SMILES attributes with unclear role).

## 5. Conclusions

- Optimal descriptors calculated by the Monte Carlo method can provide reasonable prediction for the carcinogenicity log(TD50).
- Blocking of rare SMILES attributes can improve statistical quality of the predicting. Splits into subtraining, calibration and test sets, as well splits into the training and test sets have influence to statistical characteristics of the models. In our case, in three splits examined in this study these characteristics are similar.
- The correlation balance, i.e., the **[Subtraining-Calibration-Test] system** gave models which are better in comparison with models obtained with the more traditional **[Training-Test] system.**

## Supplementary Materials

**Table 1.** Three splits into subtraining, calibration, and test sets, which were studied.

	**CAS No Split1**	**CAS No Split2**	**CAS No Split3**
	**Subtraining set**		
1.	75-07-0	75-07-0	75-07-0
2.	60-35-5	60-35-5	60-35-5
3.	34627-78-6	53-96-3	53-96-3
4.	4075-79-0	7008-42-6	7008-42-6
5.	53-96-3	79-06-1	79-06-1
6.	79-06-1	3688-53-7	107-13-1
7.	107-13-1	81-49-2	3688-53-7
8.	3688-53-7	3775-55-1	81-49-2
9.	81-49-2	99-57-0	3775-55-1
10.	3775-55-1	117-79-3	99-57-0
11.	712-68-5	97-56-3	121-88-0
12.	99-57-0	10589-74-9	117-79-3
13.	121-88-0	140-57-8	2432-99-7
14.	117-79-3	1912-24-9	10589-74-9
15.	60142-96-3	115-02-6	115-02-6
16.	2432-99-7	17967-53-9	17967-53-9
17.	10589-74-9	50-32-8	71-43-2
18.	17967-53-9	3296-90-0	92-87-5
19.	30516-87-1	542-88-1	50-32-8
20.	71-43-2	2475-45-8	14504-15-5
21.	92-87-5	75-27-4	2475-45-8
22.	50-32-8	51333-22-3	74-96-4
23.	14504-15-5	3068-88-0	3068-88-0
24.	3296-90-0	63-25-2	63-25-2
25.	85-68-7	56-23-5	56-23-5
26.	3068-88-0	120-80-9	60391-92-6
27.	331-39-5	305-03-3	305-03-3
28.	63-25-2	77439-76-0	37087-94-8
29.	56-23-5	37087-94-8	5131-60-2
30.	305-03-3	95-83-0	75-88-7
31.	37087-94-8	150-68-5	50892-23-4
32.	75-88-7	10473-70-8	108-90-7
33.	50892-23-4	1897-45-6	107-30-2
34.	65089-17-0	102-50-1	150-68-5
35.	108-90-7	80-08-0	126-99-8
36.	107-30-2	50-29-3	1897-45-6
37.	150-68-5	53-43-0	102-50-1
38.	126-99-8	853-23-6	120-71-8
39.	1897-45-6	63019-65-8	80-08-0
40.	102-50-1	16338-97-9	853-23-6
41.	120-71-8	720-69-4	16338-97-9
42.	1163-19-5	95-80-7	720-69-4
43.	853-23-6	96-12-8	96-12-8
44.	16338-97-9	10318-26-0	10318-26-0
45.	720-69-4	106-93-4	106-93-4
46.	4106-66-5	1717-00-6	106-46-7
47.	96-12-8	107-06-2	107-06-2
48.	10318-26-0	62-73-7	101-90-6
49.	106-93-4	56-53-1	3276-41-3
50.	7572-29-4	101-90-6	119-84-6
51.	106-46-7	5803-51-0	5803-51-0
52.	105-55-5	59-35-8	91-93-0
53.	3276-41-3	55738-54-0	60-11-7
54.	91-93-0	121-69-7	59-35-8
55.	4164-28-7	26049-69-4	513-37-1
56.	513-37-1	513-37-1	106-89-8
57.	106-89-8	106-89-8	150-69-6
58.	150-69-6	140-88-5	16301-26-1
59.	16301-26-1	64-17-5	57497-29-7
60.	75-21-8	16301-26-1	75-21-8
61.	117-81-7	57497-29-7	86386-73-4
62.	110559-84-7	75-21-8	69112-98-7
63.	86386-73-4	96724-44-6	110-00-9
64.	69112-98-7	86386-73-4	67730-11-4
65.	93957-54-1	363-17-7	56-40-6
66.	98-01-1	3570-75-0	87-68-3
67.	56-40-6	110-00-9	319-84-6
68.	319-84-6	98-01-1	67-72-1
69.	67-72-1	67730-11-4	26049-70-7
70.	18774-85-1	56-40-6	122-66-7
71.	26049-70-7	87-68-3	53-95-2
72.	122-66-7	67-72-1	129-43-1
73.	53-95-2	680-31-9	96724-45-7
74.	129-43-1	26049-70-7	13743-07-2
75.	96724-45-7	53-95-2	71752-70-0
76.	71752-70-0	84545-30-2	100643-96-7
77.	100643-96-7	100643-96-7	76180-96-6
78.	76180-96-6	76180-96-6	115-11-7
79.	115-11-7	15503-86-3	542-56-3
80.	542-56-3	115-11-7	54-85-3
81.	303-34-4	542-56-3	303-34-4
82.	76956-02-0	54-85-3	108-78-1
83.	148-82-3	303-34-4	148-82-3
84.	149-30-4	76956-02-0	149-30-4
85.	5834-17-3	108-78-1	934-00-9
86.	934-00-9	148-82-3	298-81-7
87.	298-81-7	60-56-0	598-55-0
88.	598-55-0	5834-17-3	55-80-1
89.	21638-36-8	298-81-7	21638-36-8
90.	63412-06-6	1634-04-4	63412-06-6
91.	598-57-2	21340-68-1	14026-03-0
92.	33868-17-6	21638-36-8	598-57-2
93.	443-48-1	63412-06-6	76014-81-8
94.	39801-14-4	14026-03-0	64091-91-4
95.	50-07-7	76014-81-8	90-94-8
96.	3771-19-5	64091-91-4	2385-85-5
97.	2243-62-1	90-94-8	39801-14-4
98.	139-94-6	39801-14-4	50-07-7
99.	99-59-2	50-07-7	58139-48-3
100.	2122-86-3	58139-48-3	2243-62-1
101.	2578-75-8	389-08-2	139-94-6
102.	53757-28-1	2243-62-1	99-59-2
103.	24554-26-5	91-59-8	91-23-6
104.	600-24-8	139-94-6	600-24-8
105.	1836-75-5	99-59-2	1836-75-5
106.	607-57-8	59-87-0	607-57-8
107.	75-52-5	75198-31-1	555-84-0
108.	38777-13-8	36133-88-7	38777-13-8
109.	83335-32-4	4812-22-0	83335-32-4
110.	89911-78-4	555-84-0	89911-79-5
111.	96806-35-8	51-75-2	89911-78-4
112.	56222-35-6	38777-13-8	96806-35-8
113.	760-60-1	83335-32-4	760-60-1
114.	937-25-7	89911-78-4	937-25-7
115.	75881-22-0	96806-35-8	13256-11-6
116.	38347-74-9	760-60-1	75881-22-0
117.	64005-62-5	937-25-7	38347-74-9
118.	1133-64-8	13256-11-6	91308-70-2
119.	51542-33-7	38347-74-9	1133-64-8
120.	60599-38-4	1133-64-8	60599-38-4
121.	62-75-9	55-18-5	62-75-9
122.	156-10-5	62-75-9	156-10-5
123.	10595-95-6	156-10-5	20917-49-1
124.	20917-49-1	42579-28-2	42579-28-2
125.	42579-28-2	86451-37-8	86451-37-8
126.	86451-37-8	70415-59-7	70415-59-7
127.	26921-68-6	16219-98-0	55984-51-5
128.	70415-59-7	59-89-2	16219-98-0
129.	16219-98-0	5632-47-3	614-00-6
130.	614-00-6	930-55-2	59-89-2
131.	59-89-2	81795-07-5	5632-47-3
132.	26541-51-5	3096-50-2	100-75-4
133.	611-23-4	101-80-4	930-55-2
134.	303-47-9	60102-37-6	26541-51-5
135.	3096-50-2	62-44-2	611-23-4
136.	60102-37-6	60-80-0	303-47-9
137.	62-44-2	77-09-8	3096-50-2
138.	77-09-8	7227-91-0	77-09-8
139.	7227-91-0	842-07-9	7227-91-0
140.	90-43-7	50-33-9	50-33-9
141.	51-03-6	122-60-1	90-43-7
142.	29069-24-7	51-03-6	51-03-6
143.	50-24-8	1955-45-9	1955-45-9
144.	671-16-9	29069-24-7	29069-24-7
145.	1120-71-4	816-57-9	57-57-8
146.	57-57-8	75-56-9	13010-07-6
147.	13010-07-6	599-79-1	81-54-9
148.	51-52-5	2318-18-5	2425-85-6
149.	2425-85-6	10048-13-2	480-54-6
150.	480-54-6	18883-66-4	2318-18-5
151.	94-59-7	96-09-3	10048-13-2
152.	2318-18-5	95-06-7	18883-66-4
153.	10048-13-2	23031-25-6	95-06-7
154.	18883-66-4	127-18-4	116-14-3
155.	96-09-3	116-14-3	109-99-9
156.	95-06-7	509-14-8	509-14-8
157.	127-18-4	139-65-1	52-24-4
158.	109-99-9	62-56-6	139-65-1
159.	62-56-6	68-76-8	88-19-7
160.	88-19-7	538-23-8	68-76-8
161.	68-76-8	88-06-2	76-25-5
162.	76-25-5	96-18-4	75-25-2
163.	75-25-2	2489-77-2	137-17-7
164.	51-79-6	51-79-6	51-79-6
165.	88-12-0	593-60-2	88-12-0
	**Calibration set**		
1.	18523-69-8	18523-69-8	18523-69-8
2.	7008-42-6	34627-78-6	34627-78-6
3.	2835-39-4	4075-79-0	4075-79-0
4.	760-56-5	107-13-1	760-56-5
5.	82-28-0	1162-65-8	82-28-0
6.	119-34-6	760-56-5	712-68-5
7.	121-66-4	82-28-0	119-34-6
8.	97-56-3	712-68-5	121-66-4
9.	61-82-5	119-34-6	97-56-3
10.	115-02-6	60142-96-3	60142-96-3
11.	103-33-3	61-82-5	61-82-5
12.	88133-11-3	25843-45-2	1912-24-9
13.	271-89-6	30516-87-1	103-33-3
14.	542-88-1	88133-11-3	25843-45-2
15.	2475-45-8	71-43-2	30516-87-1
16.	75-27-4	92-87-5	88133-11-3
17.	74-96-4	271-89-6	271-89-6
18.	51333-22-3	14504-15-5	3296-90-0
19.	106-99-0	2784-94-3	542-88-1
20.	75-65-0	106-99-0	2784-94-3
21.	60391-92-6	75-65-0	51333-22-3
22.	115-28-6	115-28-6	106-99-0
23.	101-79-1	101-79-1	75-65-0
24.	77439-76-0	5131-60-2	85-68-7
25.	5131-60-2	75-88-7	115-28-6
26.	593-70-4	65089-17-0	101-79-1
27.	54749-90-5	107-30-2	77439-76-0
28.	52214-84-3	126-99-8	65089-17-0
29.	637-07-0	52214-84-3	593-70-4
30.	123-73-9	637-07-0	10473-70-8
31.	50-18-0	120-71-8	52214-84-3
32.	80-08-0	123-73-9	637-07-0
33.	50-29-3	50-18-0	123-73-9
34.	63019-65-8	1163-19-5	50-18-0
35.	95-80-7	4106-66-5	50-29-3
36.	56654-52-5	56654-52-5	1163-19-5
37.	1717-00-6	7572-29-4	63019-65-8
38.	91-94-1	106-46-7	95-80-7
39.	107-06-2	91-94-1	56654-52-5
40.	62-73-7	111-46-6	1717-00-6
41.	685-91-6	3276-41-3	7572-29-4
42.	111-46-6	119-84-6	91-94-1
43.	56-53-1	94-58-6	62-73-7
44.	119-84-6	91-93-0	685-91-6
45.	94-58-6	65176-75-2	111-46-6
46.	5803-51-0	60-11-7	56-53-1
47.	65176-75-2	551-92-8	94-58-6
48.	60-11-7	123-91-1	65176-75-2
49.	59-35-8	57-63-6	551-92-8
50.	551-92-8	150-69-6	26049-69-4
51.	26049-69-4	100-41-4	123-91-1
52.	123-91-1	96-45-7	13256-06-9
53.	13256-06-9	117-81-7	57-63-6
54.	57-63-6	110559-84-7	140-88-5
55.	140-88-5	38434-77-4	64-17-5
56.	64-17-5	69112-98-7	100-41-4
57.	57497-29-7	93957-54-1	96-45-7
58.	100-41-4	556-52-5	117-81-7
59.	96-45-7	517-28-2	96724-44-6
60.	96724-44-6	118-74-1	110559-84-7
61.	38434-77-4	319-84-6	38434-77-4
62.	363-17-7	122-66-7	363-17-7
63.	110-00-9	306-83-2	93957-54-1
64.	67730-11-4	129-43-1	3570-75-0
65.	556-52-5	33389-36-5	556-52-5
66.	517-28-2	71752-70-0	517-28-2
67.	118-74-1	5208-87-7	118-74-1
68.	87-68-3	21416-87-5	680-31-9
69.	680-31-9	53-86-1	26049-68-3
70.	26049-68-3	86315-52-8	306-83-2
71.	306-83-2	78-59-1	33389-36-5
72.	13743-07-2	3778-73-2	5208-87-7
73.	33389-36-5	143-50-0	21416-87-5
74.	5208-87-7	5989-27-5	84545-30-2
75.	84545-30-2	77500-04-0	53-86-1
76.	53-86-1	149-30-4	15503-86-3
77.	15503-86-3	57-39-6	86315-52-8
78.	86315-52-8	934-00-9	78-59-1
79.	54-85-3	150-76-5	3778-73-2
80.	78-59-1	598-55-0	143-50-0
81.	3778-73-2	55-80-1	5989-27-5
82.	143-50-0	70-25-7	76956-02-0
83.	5989-27-5	129-15-7	57-39-6
84.	108-78-1	63642-17-1	60-56-0
85.	57-39-6	452-86-8	150-76-5
86.	60-56-0	56-49-5	1634-04-4
87.	150-76-5	101-14-4	70-25-7
88.	1634-04-4	838-88-0	129-15-7
89.	21340-68-1	598-57-2	63642-17-1
90.	70-25-7	33868-17-6	98-85-1
91.	63642-17-1	443-48-1	452-86-8
92.	98-85-1	3771-19-5	56-49-5
93.	452-86-8	139-13-9	101-14-4
94.	56-49-5	2578-75-8	838-88-0
95.	101-14-4	531-82-8	33868-17-6
96.	838-88-0	24554-26-5	443-48-1
97.	101-61-1	91-23-6	315-22-0
98.	76014-81-8	98-95-3	3771-19-5
99.	64091-91-4	600-24-8	389-08-2
100.	2385-85-5	1836-75-5	59-87-0
101.	315-22-0	607-57-8	75198-31-1
102.	58139-48-3	67-20-9	2122-86-3
103.	389-08-2	75-52-5	36133-88-7
104.	91-59-8	551-88-2	2578-75-8
105.	139-13-9	5522-43-0	24554-26-5
106.	59-87-0	607-35-2	4812-22-0
107.	75198-31-1	16813-36-8	602-87-9
108.	36133-88-7	89911-79-5	98-95-3
109.	4812-22-0	92177-50-9	67-20-9
110.	602-87-9	56222-35-6	51-75-2
111.	91-23-6	55090-44-3	75-52-5
112.	98-95-3	75881-20-8	551-88-2
113.	67-20-9	75881-22-0	5522-43-0
114.	555-84-0	684-93-5	607-35-2
115.	51-75-2	55556-92-8	16813-36-8
116.	551-88-2	82018-90-4	92177-50-9
117.	607-35-2	75881-18-4	75896-33-2
118.	16813-36-8	91308-70-2	56222-35-6
119.	89911-79-5	91308-69-9	55090-44-3
120.	92177-50-9	51542-33-7	75881-20-8
121.	96806-34-7	60599-38-4	684-93-5
122.	55090-44-3	924-16-3	55556-92-8
123.	13256-11-6	1116-54-7	82018-90-4
124.	684-93-5	621-64-7	75881-18-4
125.	92177-49-6	10595-95-6	91308-69-9
126.	55556-92-8	614-95-9	64005-62-5
127.	82018-90-4	20917-49-1	51542-33-7
128.	75881-18-4	26921-68-6	1116-54-7
129.	91308-70-2	55984-51-5	55-18-5
130.	91308-69-9	614-00-6	621-64-7
131.	1116-54-7	68107-26-6	10595-95-6
132.	55-18-5	78246-24-9	26921-68-6
133.	621-64-7	303-47-9	78246-24-9
134.	55984-51-5	14698-29-4	14698-29-4
135.	68107-26-6	13752-51-7	101-80-4
136.	78246-24-9	1825-21-4	13752-51-7
137.	5632-47-3	50-24-8	60102-37-6
138.	14698-29-4	671-16-9	62-44-2
139.	101-80-4	1120-71-4	842-07-9
140.	13752-51-7	57-57-8	122-60-1
141.	1825-21-4	13010-07-6	50-24-8
142.	842-07-9	51-52-5	671-16-9
143.	50-33-9	81-54-9	1120-71-4
144.	122-60-1	2425-85-6	816-57-9
145.	1955-45-9	127-47-9	51-52-5
146.	816-57-9	480-54-6	127-47-9
147.	81-54-9	18559-94-9	18559-94-9
148.	127-47-9	533-31-3	533-31-3
149.	18559-94-9	77-46-3	96-09-3
150.	599-79-1	811-97-2	77-46-3
151.	533-31-3	40548-68-3	127-18-4
152.	77-46-3	109-99-9	811-97-2
153.	23031-25-6	52-24-4	40548-68-3
154.	116-14-3	62-55-5	62-55-5
155.	40548-68-3	789-61-7	789-61-7
156.	509-14-8	141-90-2	141-90-2
157.	52-24-4	88-19-7	62-56-6
158.	62-55-5	76-25-5	88-06-2
159.	789-61-7	75-25-2	42011-48-3
160.	141-90-2	137-17-7	95-63-6
161.	137-17-7	95-63-6	2489-77-2
162.	95-63-6	55-63-0	55-63-0
163.	55-63-0	126-72-7	126-72-7
164.	126-72-7	66-22-8	66-22-8
165.	108-05-4	108-05-4	108-05-4
166.	75-02-5	75-02-5	75-02-5
167.	2832-40-8	2832-40-8	2832-40-8
	**Test set**		
1.	29611-03-8	29611-03-8	29611-03-8
2.	1162-65-8	57-06-7	1162-65-8
3.	57-06-7	2835-39-4	57-06-7
4.	38514-71-5	38514-71-5	2835-39-4
5.	140-57-8	121-88-0	38514-71-5
6.	1912-24-9	121-66-4	140-57-8
7.	25843-45-2	2432-99-7	33372-39-3
8.	33372-39-3	103-33-3	75-27-4
9.	2784-94-3	33372-39-3	869-01-2
10.	869-01-2	74-96-4	331-39-5
11.	120-80-9	85-68-7	120-80-9
12.	95-83-0	869-01-2	95-83-0
13.	10473-70-8	331-39-5	54749-90-5
14.	117-10-2	60391-92-6	117-10-2
15.	1192-28-5	50892-23-4	1192-28-5
16.	53-43-0	108-90-7	53-43-0
17.	79-43-6	593-70-4	4106-66-5
18.	101-90-6	54749-90-5	79-43-6
19.	55738-54-0	117-10-2	105-55-5
20.	121-69-7	1192-28-5	55738-54-0
21.	106-88-7	79-43-6	121-69-7
22.	13073-35-3	685-91-6	4164-28-7
23.	398-32-3	105-55-5	106-88-7
24.	32852-21-4	4164-28-7	13073-35-3
25.	3570-75-0	13256-06-9	398-32-3
26.	67730-10-3	106-88-7	32852-21-4
27.	26049-71-8	13073-35-3	98-01-1
28.	21416-87-5	398-32-3	67730-10-3
29.	77500-04-0	32852-21-4	18774-85-1
30.	55-80-1	67730-10-3	26049-71-8
31.	129-15-7	18774-85-1	77500-04-0
32.	14026-03-0	26049-71-8	5834-17-3
33.	90-94-8	26049-68-3	21340-68-1
34.	531-82-8	96724-45-7	101-61-1
35.	51325-35-0	13743-07-2	91-59-8
36.	62-23-7	98-85-1	139-13-9
37.	5522-43-0	101-61-1	53757-28-1
38.	75896-33-2	2385-85-5	531-82-8
39.	75881-20-8	315-22-0	51325-35-0
40.	88208-16-6	2122-86-3	62-23-7
41.	91308-71-3	53757-28-1	96806-34-7
42.	53609-64-6	51325-35-0	92177-49-6
43.	924-16-3	602-87-9	88208-16-6
44.	40580-89-0	62-23-7	91308-71-3
45.	614-95-9	96806-34-7	53609-64-6
46.	100-75-4	75896-33-2	924-16-3
47.	930-55-2	92177-49-6	40580-89-0
48.	81795-07-5	88208-16-6	614-95-9
49.	60-80-0	91308-71-3	68107-26-6
50.	75-56-9	64005-62-5	81795-07-5
51.	22571-95-5	53609-64-6	1825-21-4
52.	811-97-2	40580-89-0	60-80-0
53.	139-65-1	100-75-4	75-56-9
54.	538-23-8	26541-51-5	94-59-7
55.	88-06-2	611-23-4	599-79-1
56.	96-18-4	90-43-7	22571-95-5
57.	42011-48-3	94-59-7	23031-25-6
58.	2489-77-2	22571-95-5	538-23-8
59.	66-22-8	42011-48-3	96-18-4
60.	593-60-2	75-01-4	593-60-2
61.	75-01-4	88-12-0	75-01-4

**Table 2.** Experimental and calculated with Eq. 3 log(TD_50_): split1, limS=4, first probe of the Monte Carlo method optimization.

**CAS No**	**SMILES**	**DCW(4)**	**Expr**	**Calc**
	**Subtraining set**			
75-07-0	CC=O	−1.6442255	−0.541	−0.782
60-35-5	CC(N)=O	2.4339941	−0.484	−0.326
34627-78-6	CC(=O)OC(C=C)c1ccc2OCOc2c1	8.9723429	0.945	0.405
4075-79-0	O=C(C)Nc1ccc(cc1)c2ccccc2	16.8254890	2.253	1.283
53-96-3	CC(=O)NC1C=CC2=C3C=CC=CC3=CC2=C1	23.7041967	2.263	2.052
79-06-1	C=CC(N)=O	6.1553307	1.278	0.090
107-13-1	C=CC#N	0.4363647	0.497	−0.549
3688-53-7	O=[N+]([O−])c2ccc(/C=C(\c1ccco1)C(N)=O)o2	19.7219116	0.926	1.607
81-49-2	O=C2c1ccccc1C(=O)c3c2c(N)c(Br)cc3Br	10.6082785	0.918	0.588
3775-55-1	Nc1nnc(o1)c2oc(cc2)[N+]([O−])=O	19.5286009	1.728	1.585
712-68-5	Nc1nnc(s1)c2oc(cc2)[N+]([O−])=O	21.4765044	2.506	1.803
99-57-0	Nc1cc(ccc1O)[N+]([O−])=O	8.7582849	−0.736	0.381
121-88-0	Nc1ccc(cc1O)[N+]([O−])=O	8.7582849	0.143	0.381
117-79-3	Nc2ccc3C(=O)c1ccccc1C(=O)c3c2	10.4267880	0.344	0.568
60142-96-3	NCC1(CC(=O)O)CCCCC1	−3.0738151	−1.533	−0.942
2432-99-7	O=C(O)CCCCCCCCCCN	−2.3822437	−0.737	−0.864
10589-74-9	CCCCCN(N=O)C(N)=O	28.9999825	2.462	2.644
17967-53-9	CC(C)[N+](\[O−])=N/C(C)C	41.1617896	4.686	4.004
30516-87-1	CC1=CN(C(=O)NC1=O)C2CC(/N=[N+]=[N−])C(CO)O2	−2.1058355	−1.637	−0.834
71-43-2	c1ccccc1	3.2902364	−0.335	−0.230
92-87-5	Nc1ccc(cc1)c2ccc(N)cc2	15.6117993	2.027	1.147
50-32-8	c1cc2c3ccc4cccc5ccc(cc2cc1)c3c45	28.9921674	2.421	2.643
14504-15-5	NC(=O)Cc2c([O−])on[n+]2Cc1ccccc1	4.7178230	−0.260	−0.071
3296-90-0	OCC(CBr)(CBr)CO	10.1582969	0.373	0.538
85-68-7	O=C(OCc1ccccc1)c2ccccc2C(=O)OCCCC	9.6647886	−0.522	0.482
3068-88-0	O=C1CC(C)O1	7.0229065	0.795	0.187
331-39-5	Oc1ccc(/C=C/C(=O)O)cc1O	2.7387186	−0.217	−0.292
63-25-2	CNC(=O)Oc2cccc1ccccc12	12.8497971	1.154	0.839
56-23-5	ClC(Cl)(Cl)Cl	12.2869593	1.827	0.776
305-03-3	O=C(O)CCCc1ccc(cc1)N(CCCl)CCCl	26.8564822	2.531	2.404
37087-94-8	CC1CC(C)CN(C1)S(=O)(=O)c2cc(C(=O)O)c(Cl)cc2	22.7615129	1.835	1.947
75-88-7	ClCC(F)(F)F	11.1755831	0.133	0.651
50892-23-4	Cc2cccc(Nc1cc(Cl)nc(SCC(=O)O)n1)c2C	19.0894905	1.871	1.536
65089-17-0	Cc2cccc(Nc1cc(Cl)nc(SCC(=O)NCCO)n1)c2C	17.8867308	1.752	1.402
108-90-7	Clc1ccccc1	0.8393418	−0.341	−0.504
107-30-2	COCCl	21.7058832	1.166	1.829
150-68-5	Clc1ccc(NC(=O)N(C)C)cc1	7.6668178	0.181	0.259
126-99-8	C=C(Cl)C=C	0.3865032	−0.150	−0.555
1897-45-6	Clc1c(C#N)c(Cl)c(C#N)c(Cl)c1Cl	−1.4730550	−0.931	−0.763
102-50-1	Nc1ccc(OC)cc1C	3.4796799	−0.535	−0.209
120-71-8	Nc1cc(C)ccc1OC	6.7795818	0.146	0.160
1163-19-5	Brc2c(Oc1c(Br)c(Br)c(Br)c(Br)c1Br)c(Br)c(Br)c(Br)c2 Br	0.8059877	−0.542	−0.508
853-23-6	CC(=O)OC2CCC3(C)C4CCC1(C)C(CCC1=O)C4CC=C 3C2	23.1543517	1.022	1.991
16338-97-9	C=CCN(CC=C)N=O	26.5787909	0.571	2.373
720-69-4	O=[N+]([O−])c1ccc(o1)c2nc(N)nc(N)n2	15.5122743	2.114	1.136
4106-66-5	Nc1ccc2c3ccccc3oc2c1	18.8006239	1.869	1.504
96-12-8	BrC(CBr)CCl	24.2664740	2.960	2.115
10318-26-0	OC(C(O)CBr)C(O)C(O)CBr	21.0738102	1.566	1.758
106-93-4	BrCCBr	13.4511485	2.092	0.906
7572-29-4	ClC#CCl	24.1260754	1.423	2.099
106-46-7	Clc1ccc(Cl)cc1	4.3705653	−0.642	−0.109
105-55-5	CCNC(=S)NCC	12.9520877	0.741	0.850
3276-41-3	O=NN1CC=CCO1	17.2055032	0.100	1.325
91-93-0	COc1cc(ccc1/N=C=O)c2ccc(\N=C=O)c(OC)c2	1.4504491	−0.740	−0.436
4164-28-7	CN(C)[N+]([O−])=O	20.1348499	2.217	1.653
513-37-1	C/C(C)=C\Cl	19.4680508	0.455	1.578
106-89-8	ClCC1CO1	15.6927349	1.495	1.156
150-69-6	CCOc1ccc(cc1)NC(N)=O	4.9437759	−0.474	−0.045
16301-26-1	[O−]\[N+](CC)=N\CC	29.8573580	3.667	2.740
75-21-8	C1CO1	4.1677964	0.316	−0.132
117-81-7	CCC(CCCC)COC(=O)c1ccccc1C(=O)OCC(CC)CCCC	−2.5987356	−0.263	−0.889
110559-84-7	O=C(NCC(C)=O)N(CC)N=O	25.1884351	2.981	2.218
86386-73-4	OC(Cn1cncn1)(Cn2cncn2)c3ccc(F)cc3F	6.0321942	0.579	0.076
69112-98-7	NC(=O)N(CCF)N=O	24.3033326	3.034	2.119
93957-54-1	O=C(O)CC(O)CC(O)/C=C/c2c(c1ccccc1n2C(C)C)c3ccc (F)cc3	14.6992645	0.517	1.045
98-01-1	O=Cc1ccco1	6.3091859	−0.852	0.107
56-40-6	NCC(=O)O	−1.3873663	−2.534	−0.753
319-84-6	ClC1C(Cl)C(Cl)C(Cl)C(Cl)C1Cl	18.2269649	1.414	1.440
67-72-1	ClC(Cl)(Cl)C(Cl)(Cl)Cl	14.7243688	0.631	1.048
18774-85-1	CCCCCCN(N=O)C(N)=O	28.1722194	2.529	2.552
26049-70-7	NNc1nc(cs1)c2ccc(cc2)[N+]([O−])=O	20.6520759	1.867	1.711
122-66-7	N(Nc1ccccc1)c2ccccc2	18.1209496	1.518	1.428
53-95-2	CC(=O)N(O)C1C=CC2=C3C=CC=CC3=CC2=C1	23.9896850	2.384	2.084
129-43-1	O=C3c1ccccc1C(=O)c2c3cccc2O	2.8757163	0.380	−0.277
96724-45-7	O=C(NCC)N(N=O)CCO	21.7630490	2.458	1.835
71752-70-0	O=C(N)N(N=O)CCCO	17.2624478	2.177	1.332
100643-96-7	O=C2Nc1ccc(cc1C2(C)C)C=3CCC(=O)NN=3	22.0312469	2.107	1.865
76180-96-6	Nc3nc2c(ccc1ncccc12)n3C	21.3468133	2.388	1.788
115-11-7	C=C(C)C	0.3184457	−1.801	−0.562
542-56-3	CC(C)CON=O	8.3162583	0.280	0.332
303-34-4	CC(C)(O)C(O)(C(C)OC)C(=O)OCC1=CCN2CCC(OC(=O)C(\C)=C\C)C12	31.4970206	3.024	2.923
76956-02-0	OCc3nc(NCCCOc2cc(CN1CCCCC1)ccc2)n(C)n3	12.5193481	−0.125	0.802
148-82-3	O=C(O)C(N)Cc1ccc(cc1)N(CCCl)CCCl	41.9895654	3.512	4.096
149-30-4	S=C1Nc2ccccc2S1	4.9343382	−0.313	−0.046
5834-17-3	COc1cc2c3ccccc3oc2cc1N	15.3424654	0.866	1.117
934-00-9	COc1cccc(O)c1O	−1.6426353	0.459	−0.782
298-81-7	COc1c3occc3cc2C=CC(=O)Oc12	12.9007718	0.824	0.844
598-55-0	NC(=O)OC	2.7475976	0.123	−0.291
21638-36-8	O=[N+]([O−])c2ccc(/C=N/N1CC(C)NC1=O)o2	15.3657507	1.649	1.120
63412-06-6	O=C(N(C)N=O)c1ccccc1	25.3611674	1.706	2.237
598-57-2	[O−][N+](=O)CN	9.4966339	0.641	0.464
33868-17-6	N#CN(C)N=O	24.8721377	2.249	2.183
443-48-1	Cc1ncc([N+]([O−])=O)n1CCO	2.0019404	−0.501	−0.374
39801-14-4	ClC13C5(Cl)C2(Cl)C4C(Cl)(C(Cl)(Cl)C12Cl)C3(Cl)C4 (Cl)C5(Cl)Cl	23.6405290	2.544	2.045
50-07-7	NC(=O)OCC3C=1C(=O)C(N)=C(C)C(=O)C=1N4CC2 NC2C34OC	46.6793245	5.509	4.621
3771-19-5	O=C(O)C(C)(C)Oc1ccc(cc1)C3CCCc2ccccc23	15.0473711	1.451	1.084
2243-62-1	Nc2cccc1c2cccc1N	6.3106925	0.357	0.107
139-94-6	O=C(Nc1ncc(s1)[N+]([O−])=O)NCC	6.7942596	0.218	0.161
99-59-2	Nc1cc(ccc1OC)[N+]([O−])=O	15.7194316	0.494	1.159
2122-86-3	O=C1NN=C(O1)c2oc(cc2)[N+]([O−])=O	17.1616511	1.360	1.321
2578-75-8	O=C(C)Nc1nnc(s1)c2ccc(o2)[N+]([O−])=O	19.6066702	1.459	1.594
53757-28-1	[O−][N+](=O)c1ccc(o1)c2cscn2	17.5109445	1.407	1.360
24554-26-5	O=CNc1nc(cs1)c2ccc(o2)[N+]([O−])=O	18.1182161	1.750	1.428
600-24-8	CC(CC)[N+]([O−])=O	10.7916363	−0.443	0.608
1836-75-5	Clc2cc(Cl)ccc2Oc1ccc(cc1)[N+]([O−])=O	5.0065304	−0.170	−0.038
607-57-8	[O−][N+](=O)C1C=CC2=C3C=CC=CC3=CC2=C1	33.1141590	2.870	3.104
75-52-5	[O−][N+](C)=O	19.9552121	0.179	1.633
38777-13-8	CC(C)Oc1ccccc1OC(=O)N(C)N=O	30.1163221	2.816	2.769
83335-32-4	FC(F)(F)CCCN(CCCC(F)(F)F)N=O	20.6003220	2.551	1.705
89911-78-4	O=NN(CCO)CC(O)CO	23.8214739	1.439	2.065
96806-35-8	O=C(NCCCl)N(N=O)CC(C)O	30.0147037	2.380	2.758
56222-35-6	CC(O)CN(CCO)N=O	22.4227138	1.181	1.909
760-60-1	CC(C)CN(N=O)C(=O)N	24.4205969	1.487	2.132
937-25-7	O=NN(C)c1ccc(F)cc1	31.5388819	2.781	2.928
75881-22-0	CN(CCCCCCCCCC)N=O	21.5702225	2.201	1.813
38347-74-9	O=C1OCCN1N=O	16.7506150	2.479	1.275
64005-62-5	O=NN(CCCCC)C(=O)OCC	29.8114261	2.270	2.735
1133-64-8	O=NN2CCCCC2c1cccnc1	25.2879439	1.206	2.229
51542-33-7	CN(N=O)C(=O)Nc1nc2ccccc2s1	28.6323803	2.320	2.603
60599-38-4	O=C(C)CN(CC(=O)C)N=O	28.7233886	2.508	2.613
62-75-9	CN(C)N=O	28.9349431	2.888	2.637
156-10-5	O=Nc2ccc(Nc1ccccc1)cc2	18.9993753	−0.006	1.526
10595-95-6	CCN(C)N=O	32.7168392	3.244	3.060
20917-49-1	O=NN1CCCCCCC1	23.6797366	3.575	2.049
42579-28-2	O=C1NC(=O)CN1N=O	19.2961524	0.469	1.559
86451-37-8	CN(N=O)CC(O)CO	21.1388672	2.317	1.765
26921-68-6	CN(N=O)CCO	25.9729565	1.907	2.306
70415-59-7	CN(N=O)CCCO	20.2587809	1.852	1.667
16219-98-0	O=NN(C)c1ccccn1	24.9818346	2.807	2.195
614-00-6	O=NN(C)c1ccccc1	26.3316239	2.982	2.346
59-89-2	O=NN1CCOCC1	21.4019649	3.028	1.795
26541-51-5	O=NN1CCSCC1	24.3742548	1.390	2.127
611-23-4	Cc1ccccc1N=O	10.7414983	0.378	0.603
303-47-9	O=C(O)C(Cc1ccccc1)NC(=O)c2cc(Cl)c3CC(C)OC(=O) c3c2O	27.3969214	3.593	2.465
3096-50-2	CC(=O)Nc2ccc3c1ccccc1C(=O)c3c2	9.8204373	1.585	0.500
60102-37-6	CN1CCC2OC(=O)C3(CC(C)C(C)(O)C(=O)OCC(=CC1)C2=O)OC3C	22.8541422	2.617	1.957
62-44-2	CCOc1ccc(cc1)NC(C)=O	6.9861875	−0.843	0.183
77-09-8	Oc1ccc(cc1)C3(OC(=O)c2ccccc23)c4ccc(O)cc4	6.3745131	−0.452	0.115
7227-91-0	CN(C)/N=N/c1ccccc1	15.5075709	1.810	1.136
90-43-7	Oc2ccccc2c1ccccc1	4.7845818	−0.134	−0.063
51-03-6	CCCc1cc2OCOc2cc1COCCOCCOCCCC	8.6169629	−0.272	0.365
29069-24-7	ClCCN(CCCl)c1ccc(cc1)CCCC(=O)OCC(=O)C5(O)CC C4C3CCC2=CC(=O)C=CC2(C)C3C(O)CC45C	26.4791878	1.527	2.362
50-24-8	OCC(=O)C4(O)CCC3C2CCC1=CC(=O)C=CC1(C)C2C (O)CC34C	24.1947047	2.372	2.107
671-16-9	CC(C)NC(=O)c1ccc(CNNC)cc1	15.0574555	1.742	1.085
1120-71-4	O=S1(=O)CCCO1	6.0564342	1.503	0.079
57-57-8	O=C1CCO1	10.5571479	1.693	0.582
13010-07-6	N/C(=N/[N+]([O−])=O)N(CCC)N=O	36.6885548	2.126	3.504
51-52-5	S=C1NC(CCC)=CC(=O)N1	10.2034731	1.094	0.543
2425-85-6	[O−][N+](=O)c3cc(C)ccc3N\N=C1\c2ccccc2C=CC1=O	−6.2022200	−0.581	−1.292
480-54-6	O=C1OCC3=CCN2CCC(OC(=O)C(/CC(C)C1(O)CO)=C\C)C23	4.9508078	−0.390	−0.045
94-59-7	C=CCc1ccc2OCOc2c1	5.7835004	−0.434	0.048
2318-18-5	O=C1OC2CCN(C)CC=C(COC(=O)C(C)(O)C(C)C\C1=C\C)C2=O	23.6803007	2.332	2.049
10048-13-2	Oc2cccc3Oc1c4C5C=COC5Oc4cc(OC)c1C(=O)c23	35.8981610	3.329	3.415
18883-66-4	OC1OC(CO)C(O)C(O)C1NC(=O)N(C)N=O	39.1285358	2.440	3.776
96-09-3	c1ccccc1C2CO2	7.7920177	0.336	0.273
95-06-7	C=C(Cl)CSC(=S)N(CC)CC	8.2349252	0.933	0.323
127-18-4	Cl/C(Cl)=C(\Cl)Cl	15.8707394	0.215	1.176
109-99-9	C1CCCO1	3.2078794	−0.752	−0.239
62-56-6	NC(N)=S	11.0889155	−0.112	0.642
88-19-7	Cc1ccccc1S(=O)(N)=O	−2.1709891	−1.364	−0.841
68-76-8	O=C1C=C(C(=O)C(=C1N2CC2)N3CC3)N4CC4	40.6549214	4.662	3.947
76-25-5	OCC(=O)C54OC(C)(C)OC5CC3C2CCC1=CC(=O)C=C C1(C)C2(F)C(O)CC34C	43.7916285	3.914	4.298
75-25-2	BrC(Br)Br	5.8279398	−0.409	0.053
51-79-6	NC(=O)OCC	5.1255209	0.334	−0.025
88-12-0	O=C1CCCN1C=C	16.7959985	0.967	1.280
	**Calibration set**			
18523-69-8	C\C(C)=N\Nc1ncc(s1)c2ccc(o2)[N+]([O−])=O	15.7962887	1.644	1.168
7008-42-6	CN3c2c(c(cc1OC(C)(C)C=Cc12)OC)C(=O)c4ccccc34	23.9804592	2.804	2.083
2835-39-4	CC(C)CC(=O)OCC=C	4.9735733	0.063	−0.042
760-56-5	NC(=O)N(CC=C)N=O	21.9012133	2.578	1.850
82-28-0	O=C3c1ccccc1C(=O)c2c3ccc(C)c2N	17.3450396	0.603	1.341
119-34-6	O=[N+]([O−])c1cc(N)ccc1O	9.7056304	−0.302	0.487
121-66-4	[O−][N+](=O)c1cnc(N)s1	10.4559636	0.513	0.571
97-56-3	Cc2cc(/N=N/c1ccccc1C)ccc2N	14.9214206	1.746	1.070
61-82-5	Nc1nncn1	8.4764466	0.927	0.350
115-02-6	N#[N+]\C=C(/[O−])OCC(N)C(=O)O	16.6905834	2.339	1.268
103-33-3	N(=N/c1ccccc1)\c2ccccc2	13.6683807	0.879	0.930
88133-11-3	Nc1nc(c(CCOCC)c2ncnn12)c3ccccc3	6.2415469	−0.286	0.100
271-89-6	c1cccc2occc12	7.3775200	−0.555	0.227
542-88-1	ClCOCCl	26.3207801	4.507	2.345
2475-45-8	Nc3ccc(N)c2C(=O)c1c(N)ccc(N)c1C(=O)c23	8.2016002	0.235	0.319
75-27-4	BrC(Cl)Cl	15.6604646	0.354	1.153
74-96-4	BrCC	7.1144910	−0.136	0.197
51333-22-3	OCC(=O)C53OC(OC5CC2C1CCC4=CC(=O)C=CC4(C)C1C(O)CC23C)CCC	24.6288783	3.170	2.155
106-99-0	C=CC=C	−2.4847352	−0.683	−0.876
75-65-0	CC(C)(C)O	−1.1616802	0.060	−0.728
60391-92-6	O=C(N)N(N=O)CC(=O)O	22.4120388	1.533	1.908
115-28-6	ClC2(Cl)C1(Cl)C(Cl)=C(Cl)C2(Cl)C(C1C(=O)O)C(=O) O	10.5026263	0.979	0.576
101-79-1	Clc2ccc(Oc1ccc(N)cc1)cc2	13.5687773	0.767	0.919
77439-76-0	ClC=1C(=O)OC(O)C=1C(Cl)Cl	24.3423352	2.572	2.123
5131-60-2	Nc1ccc(Cl)c(N)c1	5.3139941	−0.344	−0.004
593-70-4	ClCF	10.5959992	0.396	0.587
54749-90-5	OC1OC(CO)C(O)C(O)C1NC(=O)N(CCCl)N=O	31.9896113	3.923	2.978
52214-84-3	ClC2(Cl)CC2c1ccc(OC(C)(C)C(=O)O)cc1	20.0158390	2.123	1.640
637-07-0	Clc1ccc(OC(C)(C)C(=O)OCC)cc1	3.8349646	0.157	−0.169
123-73-9	C\C=C\C=O	4.9703123	1.222	−0.042
50-18-0	O=P1(NCCCO1)N(CCCl)CCCl	19.9930944	2.072	1.637
80-08-0	Nc1ccc(cc1)S(=O)(=O)c2ccc(N)cc2	10.1590864	1.045	0.538
50-29-3	Clc1ccc(cc1)C(c2ccc(Cl)cc2)C(Cl)(Cl)Cl	9.0943378	0.622	0.419
63019-65-8	CC(=O)N(C(C)=O)C2C=CC=C1c3ccccc3C=C12	20.4197854	1.145	1.685
95-80-7	Nc1cc(N)c(C)cc1	4.6373599	1.694	−0.080
56654-52-5	O=C(NCCCC)N(CCCC)N=O	21.9450360	1.672	1.855
1717-00-6	CC(Cl)(Cl)F	−3.0596285	−1.653	−0.940
91-94-1	Nc1ccc(cc1Cl)c2ccc(N)c(Cl)c2	13.5727307	0.955	0.919
107-06-2	ClCCCl	20.7099520	1.090	1.717
62-73-7	COP(=O)(OC)O\C=C(\Cl)Cl	22.6999204	1.725	1.940
685-91-6	CCN(CC)C(C)=O	12.0480263	1.115	0.749
111-46-6	OCCOCCO	−5.3166083	−1.194	−1.192
56-53-1	Oc1ccc(cc1)C(\CC)=C(\CC)c2ccc(O)cc2	20.1502302	3.080	1.655
119-84-6	O=C1CCc2ccccc2O1	−2.0947876	−1.302	−0.832
94-58-6	CCCc1ccc2OCOc2c1	7.0532301	0.060	0.190
5803-51-0	COc2ccc(cc2/C=C/c1ccc(N)cc1)OC	18.2640447	2.549	1.444
65176-75-2	COc5c(OC)cc(O)c2c5Oc1c3C4C=COC4Oc3cc(OC)c1C 2=O	27.2013851	3.024	2.443
60-11-7	CN(C)c2ccc(/N=N/c1ccccc1)cc2	25.0188899	1.833	2.199
59-35-8	O=[N+]([O−])c1ccc(o1)c2nc(C)cc(C)n2	23.6523030	2.198	2.046
551-92-8	O=[N+]([O−])c1cnc(C)n1C	11.8799028	0.919	0.730
26049-69-4	CN(C)Nc1nc(cs1)c2ccc(o2)[N+]([O−])=O	32.0887363	2.793	2.989
123-91-1	C1COCCO1	3.8263120	−0.481	−0.170
13256-06-9	CCCCCN(CCCCC)N=O	20.8293992	1.665	1.731
57-63-6	Oc3cc4CCC2C(CCC1(C)C2CCC1(O)C#C)c4cc3	23.6026061	3.171	2.041
140-88-5	C=CC(=O)OCC	−0.4544704	−0.075	−0.649
64-17-5	CCO	−2.0986452	−2.296	−0.833
57497-29-7	[O−]\[N+](CC)=N\C	35.5286934	3.669	3.374
100-41-4	CCc1ccccc1	−0.2763213	−1.612	−0.629
96-45-7	S=C1NCCN1	15.6987509	1.099	1.157
96724-44-6	O=NN(CC)C(=O)NCCO	24.0147978	2.490	2.087
38434-77-4	N#CN(CC)N=O	27.9326312	1.430	2.525
363-17-7	FC(F)(F)C(=O)NC1C=CC2=C3C=CC=CC3=CC2=C1	21.8934039	2.233	1.850
110-00-9	c1ccco1	11.1441338	2.235	0.648
67730-11-4	Cc1cccn2c3nc(N)ccc3nc12	17.7533734	1.626	1.387
556-52-5	OCC1CO1	8.2028179	1.238	0.319
517-28-2	Oc2cc3CC4(O)COc1c(O)c(O)ccc1C4c3cc2O	1.6535029	−0.520	−0.413
118-74-1	Clc1c(Cl)c(Cl)c(Cl)c(Cl)c1Cl	18.0729350	1.868	1.422
87-68-3	Cl/C(Cl)=C(/Cl)\C(\Cl)=C(/Cl)Cl	5.0800268	0.598	−0.030
680-31-9	CN(C)P(=O)(N(C)C)N(C)C	24.8394682	3.717	2.179
26049-68-3	NNc1nc(cs1)c2oc(cc2)[N+]([O−])=O	20.4685616	1.851	1.690
306-83-2	ClC(Cl)C(F)(F)F	5.3073777	−1.190	−0.005
13743-07-2	NC(=O)N(N=O)CCO	22.3036387	2.737	1.895
33389-36-5	O=[N+]([O−])c1ccc(s1)c2nc(NCCO)c3ccccc3n2	20.5124644	2.228	1.695
5208-87-7	C=CC(O)c1ccc2OCOc2c1	5.6164087	0.986	0.030
84545-30-2	FC(F)(F)C\N=C(/N)Nc1ccn(CCCCC(N)=O)n1	−1.1528528	−0.582	−0.727
53-86-1	Clc1ccc(cc1)C(=O)n3c2ccc(cc2c(CC(=O)O)c3C)OC	20.8646978	2.493	1.735
15503-86-3	[O−][N+]13CC=C2COC(=O)[C@@](O)(CO)[C@H](C)C/C (=C\C)C(=O)OC(CC1)C23	23.0174870	2.710	1.975
86315-52-8	CS(=O)c3ccc(c1nc2cnccc2n1)c(OC)c3	12.4221377	0.610	0.791
54-85-3	O=C(NN)c1ccncc1	6.9743574	−0.039	0.182
78-59-1	O=C1C=C(C)CC(C)(C)C1	7.5076649	−0.942	0.241
3778-73-2	O=P1(NCCCl)OCCCN1CCCl	22.8638696	2.548	1.958
143-50-0	O=C2C1(Cl)C3(Cl)C5(Cl)C1(Cl)C4(Cl)C2(Cl)C3(Cl)C 4(Cl)C5(Cl)Cl	20.4516977	2.219	1.688
5989-27-5	CC1=CCC(CC1)C(C)=C	4.0566866	−0.175	−0.145
108-78-1	Nc1nc(N)nc(N)n1	3.6878686	−0.765	−0.186
57-39-6	CC1CN1P(=O)(N2CC2C)N3CC3C	27.2097154	1.684	2.444
60-56-0	S=C1NC=CN1C	14.2653106	2.001	0.997
150-76-5	Oc1ccc(OC)cc1	0.9123388	−0.724	−0.496
1634-04-4	CC(C)(C)OC	5.6530612	−0.901	0.034
21340-68-1	Clc1ccc(cc1)c2ccc(OC(C)(C)C(=O)OC)cc2	17.6806284	1.805	1.379
70-25-7	O=[N+]([O−])\N=C(\N)N(C)N=O	28.6725181	2.263	2.607
63642-17-1	NC(CCCNC(=O)N(C)N=O)C(=O)O	28.8570438	2.443	2.628
98-85-1	CC(O)c1ccccc1	−0.4434130	−0.574	−0.648
452-86-8	Cc1cc(O)c(O)cc1	0.8376489	−0.301	−0.504
56-49-5	Cc2ccc3cc1c5ccccc5ccc1c4CCc2c34	28.4179889	2.738	2.579
101-14-4	Nc2ccc(Cc1ccc(N)c(Cl)c1)cc2Cl	10.3247375	1.141	0.556
838-88-0	Cc2cc(Cc1ccc(N)c(C)c1)ccc2N	14.6440072	1.487	1.039
101-61-1	CN(C)c2ccc(Cc1ccc(cc1)N(C)C)cc2	19.3590719	1.191	1.566
76014-81-8	OC(CCCN(C)N=O)c1cccnc1	30.8490216	3.308	2.851
64091-91-4	O=C(CCCN(C)N=O)c1cccnc1	29.2949689	3.317	2.677
2385-85-5	ClC53C1(Cl)C4(Cl)C2(Cl)C1(Cl)C(Cl)(Cl)C5(Cl)C2(Cl)C3(Cl)C4(Cl)Cl	27.1109046	2.489	2.433
315-22-0	O=C1OCC3=CCN2CCC(OC(=O)C(C)C(C)(O)C1(C)O) C23	26.1356689	2.539	2.324
58139-48-3	O=[N+]([O−])c1ccc(s1)c3nc(N2CCOCC2)c4ccccc4n3	22.4025459	1.833	1.907
389-08-2	O=C(O)C2=CN(CC)c1nc(C)ccc1C2=O	2.9489805	0.063	−0.268
91-59-8	Nc1ccc2ccccc2c1	4.7774007	0.366	−0.064
139-13-9	OC(=O)CN(CC(=O)O)CC(=O)O	1.4031307	−0.967	−0.441
59-87-0	O=[N+]([O−])c1ccc(/C=N/NC(N)=O)o1	15.4159812	1.453	1.125
75198-31-1	O=[N+]([O−])c1ccc(o1)c2cnc3ccccn23	16.2049151	1.227	1.214
36133-88-7	[O−][N+](=O)c1ccc(o1)c2nc(CNC(C)=O)on2	12.4300126	0.627	0.792
4812-22-0	CC\C=C(/CC)[N+]([O−])=O	13.7273230	1.174	0.937
602-87-9	[O−][N+](=O)c1ccc2CCc3cccc1c23	9.4562186	1.361	0.459
91-23-6	COc1ccccc1[N+]([O−])=O	−2.4235018	0.992	−0.869
98-95-3	[O−][N+](=O)c1ccccc1	11.5415934	0.684	0.692
67-20-9	O=[N+]([O−])c2ccc(/C=N/N1CC(=O)NC1=O)o2	9.0897766	0.165	0.418
555-84-0	O=[N+]([O−])c2ccc(/C=N/N1CCNC1=O)o2	11.4825719	1.630	0.686
51-75-2	ClCCN(C)CCCl	30.5203075	4.137	2.814
551-88-2	CCC(CC)[N+]([O−])=O	12.8577847	0.694	0.839
607-35-2	[O−][N+](=O)c1cccc2cccnc12	12.5000850	1.249	0.799
16813-36-8	O=C1NC(=O)N(N=O)CC1	21.4966956	3.163	1.805
89911-79-5	O=NN(CC(C)O)CC(O)CO	26.1835997	3.523	2.329
92177-50-9	OC(CNCC(C)=O)C(O)N=O	22.4907916	3.699	1.916
96806-34-7	O=C(NCCCl)N(N=O)CCO	28.0419947	2.740	2.537
55090-44-3	CN(CCCCCCCCCCCC)N=O	20.6335287	2.629	1.709
13256-11-6	CN(CCc1ccccc1)N=O	26.1136794	4.216	2.321
684-93-5	NC(=O)N(C)N=O	25.2656253	3.046	2.227
92177-49-6	O=C(N=O)CCNCCO	15.4212329	1.910	1.126
55556-92-8	O=NN1CC=CCC1	21.4577569	3.271	1.801
82018-90-4	FC(F)(F)CN(CC)N=O	20.7170961	1.792	1.718
75881-18-4	CC1CN(N=O)CC(C)N1C	39.3778121	3.018	3.804
91308-70-2	CC(O)CN(CC=C)N=O	26.6342790	2.216	2.380
91308-69-9	C=CCN(N=O)CCO	19.8460294	2.423	1.621
1116-54-7	OCCN(N=O)CCO	16.1731320	1.627	1.210
55-18-5	CCN(CC)N=O	25.0720908	3.586	2.205
621-64-7	CCCN(CCC)N=O	28.5592505	2.845	2.595
55984-51-5	CC(=O)CN(C)N=O	26.5154639	3.829	2.366
68107-26-6	CN(CCCCCCCCCCC)N=O	21.1018756	1.956	1.761
78246-24-9	O=NN2CCCC2c1c[n+]([O−])ccc1	21.5748823	2.344	1.814
5632-47-3	O=NN1CCNCC1	22.9448185	1.118	1.967
14698-29-4	O=C(O)C2=CN(CC)c1cc3OCOc3cc1C2=O	7.1612743	0.194	0.203
101-80-4	Nc1ccc(cc1)Oc2ccc(N)cc2	16.4624683	1.323	1.242
13752-51-7	S=C(SN1CCOCC1)N2CCOCC2	11.7032524	0.437	0.710
1825-21-4	Clc1c(OC)c(Cl)c(Cl)c(Cl)c1Cl	16.7076547	1.053	1.270
842-07-9	O=C3C=Cc1ccccc1/C3=N\Nc2ccccc2	8.1054887	0.927	0.308
50-33-9	O=C3C(CCCC)C(=O)N(c1ccccc1)N3c2ccccc2	3.0841934	−0.575	−0.253
122-60-1	c2ccc(OCC1CO1)cc2	5.8485539	0.533	0.056
1955-45-9	O=C1OCC1(C)C	4.2138276	−0.324	−0.127
816-57-9	NC(=O)N(CCC)N=O	22.6119432	1.541	1.930
81-54-9	O=C2c1ccccc1C(=O)c3c2c(O)cc(O)c3O	11.7313515	−0.423	0.713
127-47-9	CC=1CCCC(C)(C)C=1/C=CC(\C)=C\C=C\C(\C)=C\CO C(C)=O	12.3716885	0.420	0.785
18559-94-9	OCc1cc(ccc1O)C(O)CNC(C)(C)C	3.2247443	0.777	−0.238
599-79-1	O=S(=O)(Nc1ccccn1)c3ccc(N\N=C2/C=CC(=O)C(=C2) C(=O)O)cc3	2.6830187	−0.601	−0.298
533-31-3	Oc1ccc2OCOc2c1	5.4856314	−0.990	0.015
77-46-3	O=S(=O)(c1ccc(NC(C)=O)cc1)c2ccc(NC(C)=O)cc2	14.4156550	0.777	1.014
23031-25-6	Oc1cc(cc(O)c1)C(O)CNC(C)(C)C	6.0019117	−0.260	0.073
116-14-3	F/C(F)=C(\F)F	2.7326963	−0.029	−0.293
40548-68-3	O=NN1CCCCO1	18.8342347	0.679	1.508
509-14-8	O=[N+]([O−])C([N+]([O−])=O)([N+](=O)[O−])[N+]([O−])=O	19.9621805	2.642	1.634
52-24-4	S=P(N1CC1)(N2CC2)N3CC3	28.4599896	3.062	2.584
62-55-5	CC(N)=S	8.4251325	0.815	0.344
789-61-7	NC=3Nc2c(ncn2C1CC(O)C(CO)O1)C(=S)N=3	25.3320122	2.130	2.234
141-90-2	O=C1C=CNC(=S)N1	17.6865810	1.032	1.379
137-17-7	Cc1cc(C)c(N)cc1C	0.3523384	0.605	−0.559
95-63-6	Cc1cc(C)c(C)cc1	3.1538748	−1.559	−0.245
55-63-0	O=[N+]([O−])OC(CO[N+]([O−])=O)CO[N+](=O)[O−]	8.5730907	0.094	0.360
126-72-7	BrCC(Br)COP(=O)(OCC(Br)CBr)OCC(Br)CBr	21.6072056	2.260	1.818
108-05-4	CC(=O)OC=C	0.4943662	−0.598	−0.543
75-02-5	C=CF	−0.6009610	0.362	−0.665
2832-40-8	O=C2C=CC(C)=C\C2=N\Nc1ccc(NC(C)=O)cc1	5.1582118	−0.149	−0.021
	**Test set**			
29611-03-8	O=C2Oc1c4C5C=COC5Oc4cc(OC)c1C=3CCC(O)C2=3	37.2419668	5.102	3.566
1162-65-8	O=C2Oc1c4C5C=COC5Oc4cc(OC)c1C=3CCC(=O)C2 =3	37.9690087	4.991	3.647
57-06-7	C=CC\N=C=S	0.5282930	0.014	−0.539
38514-71-5	Nc1nc(cs1)c2oc(cc2)[N+]([O−])=O	16.1535617	1.558	1.208
140-57-8	CC(C)(C)c1ccc(OCC(C)OS(=O)OCCCl)cc1	6.9764303	0.539	0.182
1912-24-9	Clc1nc(NCC)nc(NC(C)C)n1	11.2226837	0.833	0.657
25843-45-2	[O−]\[N+](C)=N\C	32.4681999	3.201	3.032
33372-39-3	O=[N+]([O−])c1ccc(s1)c2nc(N(CCO)CCO)c3ccccc3n2	22.0260018	2.060	1.864
2784-94-3	CNc1ccc(cc1[N+]([O−])=O)N(CCO)CCO	−0.0656579	−0.439	−0.605
869-01-2	O=C(N)N(CCCC)N=O	25.3510763	2.448	2.236
120-80-9	Oc1ccccc1O	−0.0151194	0.114	−0.600
95-83-0	Nc1cc(Cl)ccc1N	8.8914505	−0.176	0.396
10473-70-8	Clc1ccc(NC(=O)N(C)C)cc1	7.6668178	1.512	0.259
117-10-2	Oc3cccc2C(=O)c1cccc(O)c1C(=O)c23	2.2118706	−0.009	−0.351
1192-28-5	O\N=C1\CCCC1	5.4517784	0.385	0.011
53-43-0	O=C2CCC1C3CC=C4CC(O)CCC4(C)C3CCC12C	13.1419721	0.538	0.871
79-43-6	ClC(Cl)C(=O)O	6.7872450	−0.096	0.161
101-90-6	c1ccc(cc1OCC2CO2)OCC3CO3	17.1281712	1.769	1.317
55738-54-0	CN(C)CNc2nnc(/C=C/c1ccc(o1)[N+]([O−])=O)o2	9.7862431	1.096	0.496
121-69-7	CN(C)c1ccccc1	8.7503763	−0.013	0.380
106-88-7	CCC1CO1	5.3651210	−0.484	0.002
13073-35-3	OC(=O)C(N)CCSCC	15.6037032	1.517	1.146
398-32-3	O=C(C)Nc1ccc(cc1)c2ccc(F)cc2	22.0327470	2.356	1.865
32852-21-4	O=CNNc1nc(C)cs1	6.1212428	1.038	0.086
3570-75-0	O=CNNc1nc(cs1)c2ccc(o2)[N+]([O−])=O	22.4332160	1.701	1.910
67730-10-3	Nc1ccc2nc3ccccn3c2n1	13.0355578	0.639	0.859
26049-71-8	NNc1nc(cs1)c2ccc(N)cc2	16.3483477	2.302	1.230
21416-87-5	O=C2CN(CC(C)N1CC(=O)NC(=O)C1)CC(=O)N2	10.5466160	1.399	0.581
77500-04-0	Cc1nc3c(nc1)ccc2c3nc(N)n2C	23.2794448	2.109	2.005
55-80-1	CN(C)c2ccc(/N=N/c1cc(C)ccc1)cc2	27.2082826	1.863	2.444
129-15-7	[O−][N+](=O)c3c(C)ccc2C(=O)c1ccccc1C(=O)c23	11.1318117	0.499	0.646
14026-03-0	CC1CCCCN1N=O	22.1230424	0.987	1.875
90-94-8	CN(C)c1ccc(cc1)C(=O)c2ccc(cc2)N(C)C	15.4754897	1.677	1.132
531-82-8	O=C(C)Nc1nc(cs1)c2ccc(o2)[N+]([O−])=O	22.4493722	1.153	1.912
51325-35-0	O=[N+]([O−])c1ccc(o1)c2nc(NC(C)=O)nc(NC(C)=O)n2	18.7810092	1.337	1.502
62-23-7	O=[N+]([O−])c1ccc(cc1)C(=O)O	11.6562372	−0.235	0.705
5522-43-0	[O−][N+](=O)c4ccc1ccc2cccc3ccc4c1c23	14.2090349	1.871	0.990
75896-33-2	OC1CCN(N=O)C1	27.8276236	2.162	2.513
75881-20-8	CN(CCCCCCCCCCCCCC)N=O	19.6968349	2.192	1.604
88208-16-6	O=NN(CC=C)CC(O)CO	24.1429095	2.288	2.101
91308-71-3	C=CCN(CC(=O)C)N=O	29.3465230	2.628	2.683
53609-64-6	CC(O)CN(CC(C)O)N=O	24.7848396	2.283	2.173
924-16-3	CCCCN(CCCC)N=O	30.8956853	2.360	2.856
40580-89-0	O=NN1CCCCCCCCCCCC1	15.8409546	1.290	1.173
614-95-9	O=NN(CC)C(=O)OCC	26.0539800	3.209	2.315
100-75-4	O=NN1CCCCC1	23.0864884	1.902	1.983
930-55-2	O=NN1CCCC1	21.0203400	2.098	1.752
81795-07-5	CC1SC(C)SC(C)N1N=O	22.5025937	2.600	1.918
60-80-0	O=C2C=C(C)N(C)N2c1ccccc1	13.3054506	−0.815	0.889
75-56-9	CC1CO1	11.0792966	−0.107	0.641
22571-95-5	CC(C)C(O)(C(C)O)C(=O)OCC1=CCN2CCC(OC(=O)C (\C)=C\C)C12	23.3746230	2.300	2.015
811-97-2	FCC(F)(F)F	−4.6111637	−2.467	−1.114
139-65-1	Nc1ccc(cc1)Sc2ccc(N)cc2	10.6556332	1.766	0.593
538-23-8	O=C(CCCCCCC)OC(COC(=O)CCCCCCC)COC(=O)C CCCCCC	−6.8111993	−1.067	−1.360
88-06-2	Clc1cc(Cl)cc(Cl)c1O	4.0429184	−0.312	−0.146
96-18-4	ClCC(Cl)CCl	22.1125091	2.038	1.874
42011-48-3	O=C(Nc1nc(cs1)c2ccc(o2)[N+]([O−])=O)C(F)(F)F	13.7807006	1.656	0.943
2489-77-2	CN(C)C(=S)NC	21.0749620	0.661	1.758
66-22-8	O=C1C=CNC(=O)N1	7.6978985	−0.777	0.263
593-60-2	BrC=C	6.2037631	0.762	0.095
75-01-4	C=CCl	15.1872444	1.010	1.100

## Figures and Tables

**Table 1.** The list of outliers of the QSAR models calculated with SMILES-based optimal descriptors.

**Number**	**Structure**	**CAS**	**Chemical name**
**1**	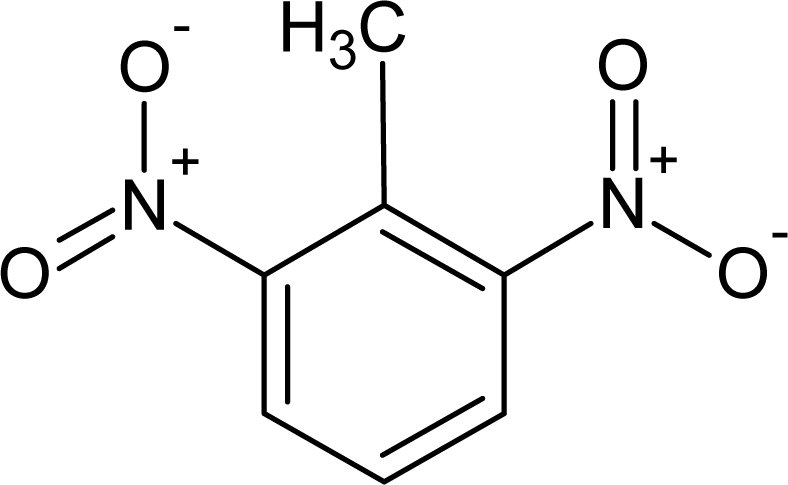	606-20-2	2,6-Dinitrotoluene
**2**	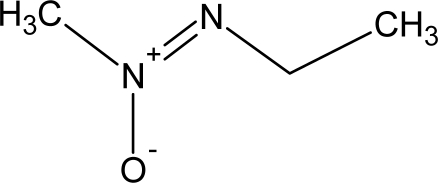	57497-34-4	*Z*-Methyl-*O,N,N*-azoxyethane
**3**	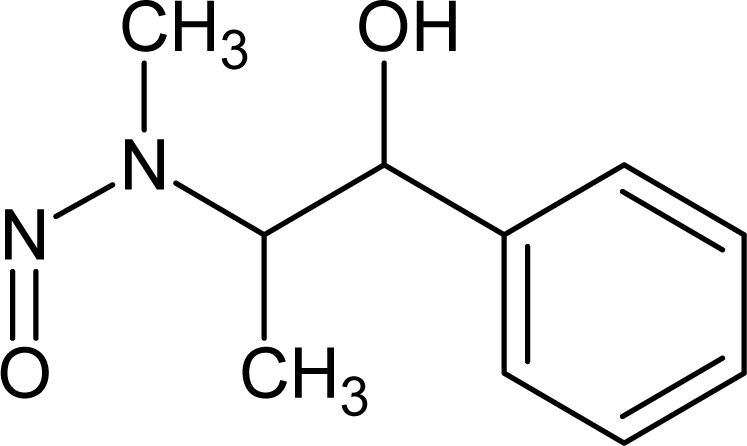	17608-59-2	*N*-Nitrosoephedrine
**4**	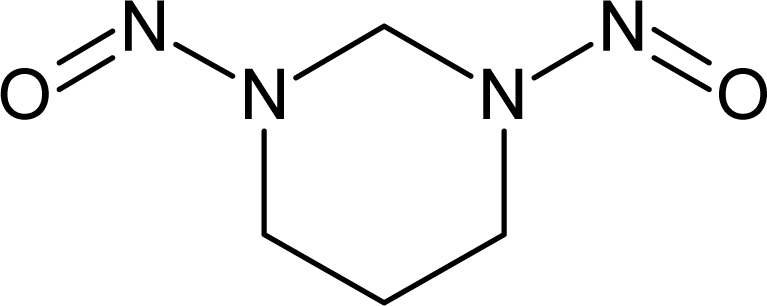	15973-99-6	Di(*N*-nitroso)-perhydropyrimidine
**5**	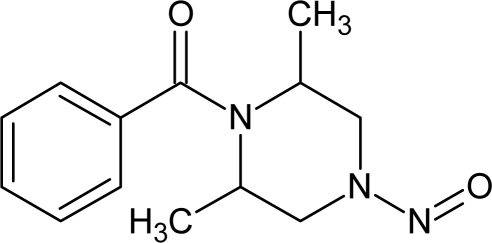	61034-40-0	1-Nitroso-4-benzoyl-3,5-dimethylpiperazine
**6**	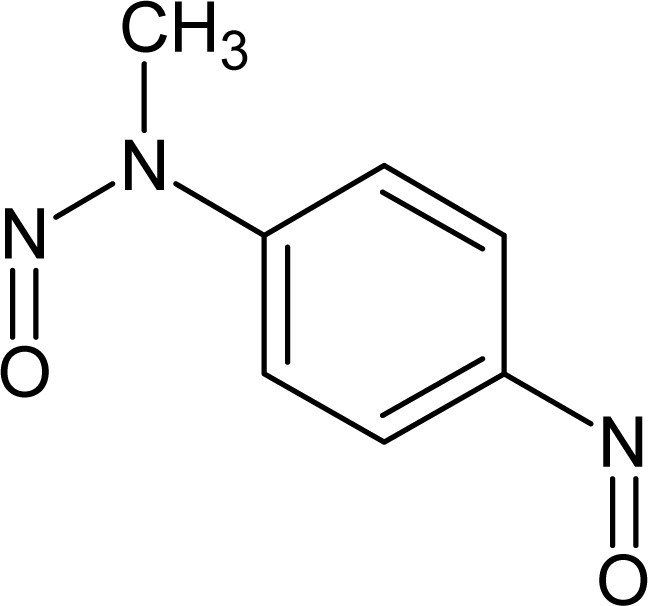	99-80-9	*N*,4-Dinitrosomethylaniline
**7**	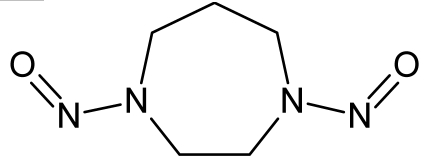	55557-00-1	*N,N*-Dinitrosohomopiperazine
**8**	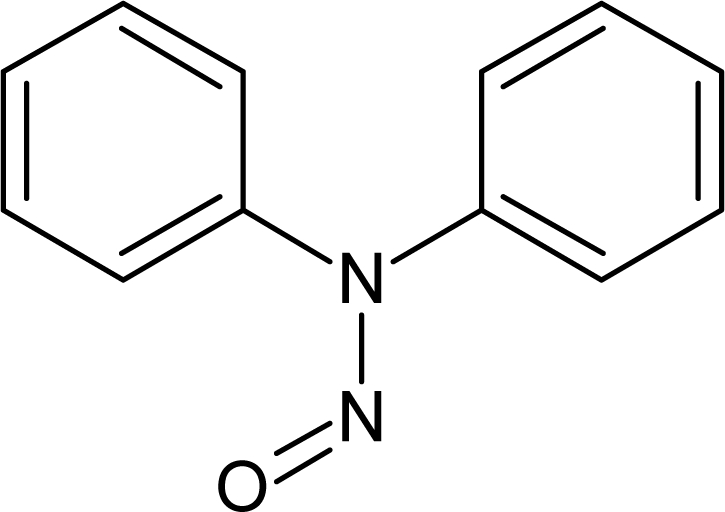	86-30-6	*N*-Nitrosodiphenylamine

**Table 2.** Example of definition of SMILES attributes (unused positions are indicated by dots).

**^1^S_k_**	**CW(^1^S_k_)**	**^2^S_k_**	**CW(^2^S_k_)**	**dC**	**CW(dC)**
C...........	−0.0156855				
O=C...........	−2.8475657	O=C.C.......	0.0	!-02........	1.2190257

SMILES=“CC=O”; CAS= 75-07-0; DCW= −1.6442255.

**Table 3.** Results of computational experiments to establish of number of epochs of the Monte Carlo optimization, Nepoch.

**[Subtraining-Calibration-Test] system**			
**Nepoch**	**r^2^_subtraining_**	**r^2^_calibration_**	**r^2^_test_**
Split-1			
5	0.5850	0.6043	0.5513
10	0.7629	0.7675	**0.7601**
15	0.7939	0.8006	0.7187
20	0.8154	0.8243	0.6827
25	0.8300	0.8262	0.6076
Split-2			
5	0.5947	0.6017	0.7347
10	0.7195	0.7190	**0.8011**
15	0.7551	0.7538	0.7870
20	0.7732	0.7719	0.7659
25	0.7839	0.7834	0.7538
Split-3			
5	0.6673	0.6303	0.6548
10	0.7656	0.7669	**0.7519**
15	0.8077	0.8080	0.7205
20	0.8436	0.8428	0.6288
25	0.8562	0.8581	0.5503
**[Training-Test] system**			
Split-1			
5	0.6255		0.6003
10	0.7761		**0.7098**
15	0.8124		0.6579
20	0.8386		0.5826
25	0.8521		0.5158
Split-2			
5	0.6028		0.7397
10	0.7396		**0.7719**
15	0.7687		0.7705
20	0.7872		0.7452
25	0.7985		0.7123
Split-3			
5	0.6328		0.6559
10	0.7682		**0.7127**
15	0.8109		0.6397
20	0.8368		0.5378
25	0.8519		0.4573

**Table 4.** Average statistical characteristics of the QSAR model of carcinogenicity (logTD_50_) for three splits into the subtraining, calibration, and test sets with the limS values of 0–10. For the best models three attempts of the Monte Carlo optimization together with average values are presented, for other models only average values are shown.

**SPLIT1**			
**Subtraining set, n=165**	**Calibration set, n=167**	**Test set, n=61**	**SA_k_ distribution**

*[Subtraining-Calibration-Test] system*												
limS	Nact	R^2^	s	F	R^2^	s	F	R^2^	s	F	W%	N_111_
0	797	0.8731	0.500	1125	0.8805	0.619	1217	0.5769	0.893	81	42	333
1	622	0.8807	0.485	1203	0.8821	0.621	1235	0.5319	0.942	67	50	314
2	407	0.8275	0.583	783	0.8268	0.703	789	0.6305	0.832	101	70	285
3	321	0.7801	0.658	579	0.7806	0.730	588	0.7102	0.732	145	79	255
**4–1**	**266**	0.7622	0.685	522	0.7620	0.734	528	0.7541	0.682	181	**82**	**217**
**4–2**		0.7593	0.689	514	0.7592	0.746	520	0.7483	0.692	175		
**4–3**		0.7643	0.682	529	0.7647	0.729	536	0.7519	0.678	179		
**average**		**0.7619**	**0.685**	**522**	**0.7619**	**0.736**	**528**	**0.7514**	**0.684**	**178**		
5	233	0.7247	0.737	429	0.7241	0.770	433	0.7387	0.711	167	85	197
6	203	0.6901	0.781	363	0.6888	0.814	365	0.7129	0.738	148	86	174
7	182	0.6704	0.806	332	0.6710	0.830	337	0.6541	0.812	112	84	153
8	164	0.6528	0.827	307	0.6530	0.844	311	0.7015	0.753	139	87	142
9	152	0.6356	0.847	284	0.6348	0.864	287	0.6378	0.822	105	84	128
10	139	0.6178	0.868	263	0.6218	0.875	271	0.6788	0.777	126	84	117

**Training set, n=332**				**Calibration set, n=0**	**Test set, n=61**	**SA_k_ distribution**

*[Training-Test] system*												
limS	Nact	R^2^	s	F	R^2^	s	F	R^2^	s	F	W%	N_101_
0	797	0.8868	0.472	2593				0.5429	1.002	71	47	376
1	777	0.8851	0.475	2542				0.5418	0.984	71	46	356
2	542	0.8602	0.524	2032				0.6042	0.910	91	61	330
3	432	0.8313	0.576	1626				0.5575	0.917	74	72	309
4	385	0.8109	0.610	1417				0.5628	0.910	76	75	289
5	344	0.8007	0.626	1327				0.5913	0.871	86	78	267
**6–1**	**312**	0.7902	0.642	1243				0.6744	0.769	122	**82**	**255**
**6–2**		0.7875	0.646	1223				0.7138	0.721	147		
**6–3**		0.7843	0.651	1200				0.6947	0.744	134		
**average**		**0.7873**	**0.647**	**1222**				**0.6943**	**0.745**	**135**		
7	288	0.7788	0.659	1162				0.6579	0.789	114	83	238
8	268	0.7659	0.678	1080				0.6677	0.777	121	85	227
9	246	0.7363	0.720	922				0.6853	0.757	129	84	207
10	234	0.7224	0.739	859				0.6909	0.750	133	84	196

**SPLIT2**			
**Subtraining set, n=165**	**Calibration set, n=167**	**Test set, n=61**	**SA_k_ distribution**

*[Subtraining-Calibration-Test] system Split2*												
limS	Nact	R^2^	s	F	R^2^	s	F	R^2^	s	F	W%	N_111_
0	797	0.8743	0.507	1134	0.8737	0.540	1142	0.4630	1.055	51	42	337
1	632	0.8740	0.507	1131	0.8736	0.551	1140	0.5003	0.995	59	51	320
2	425	0.8377	0.576	841	0.8367	0.580	846	0.5919	0.820	86	67	286
3	335	0.8048	0.632	673	0.8041	0.633	678	0.5862	0.861	84	78	261
4	284	0.7843	0.664	593	0.7842	0.663	600	0.7042	0.711	141	84	239
5	247	0.7458	0.721	478	0.7448	0.728	482	0.7627	0.671	190	87	214
**6–1**	**224**	0.7315	0.741	444	0.7314	0.748	449	0.7937	0.604	227	**84**	**189**
**6–2**		0.7234	0.752	426	0.7234	0.760	431	0.7922	0.605	225		
**6–3**		0.7384	0.731	460	0.7384	0.740	466	0.8136	0.593	258		
**average**		**0.7311**	**0.741**	**444**	**0.7310**	**0.749**	**449**	**0.7998**	**0.600**	**236**		
7	195	0.6978	0.786	376	0.7007	0.781	386	0.7318	0.657	161	84	164
8	178	0.6878	0.799	359	0.6880	0.801	364	0.7223	0.682	153	82	146
9	158	0.6659	0.826	325	0.6692	0.831	334	0.7104	0.709	145	84	133
10	149	0.6472	0.849	299	0.6550	0.847	313	0.6970	0.723	136	84	125

**Training set, n=332**	**Calibration set, N=0**	**Test set, n=61**	**SA_k_ distribution**

*[Training-Test] system Split2*												
limS	Nact	R^2^	s	F	R^2^	s	F	R^2^	s	F	W%	N_101_
0	797	0.8922	0.468	2734				0.4665	1.013	52	47	372
1	785	0.8950	0.462	2815				0.4711	1.029	53	46	360
2	546	0.8740	0.506	2290				0.5329	0.887	67	61	335
3	442	0.8456	0.561	1807				0.5767	0.845	81	71	315
4	388	0.8194	0.606	1497				0.6130	0.805	94	76	296
5	350	0.8122	0.618	1428				0.5802	0.873	82	79	278
6	321	0.8103	0.621	1412				0.6074	0.840	92	83	267
7	287	0.7848	0.662	1204				0.6689	0.753	120	86	247
8	263	0.7594	0.700	1042				0.7345	0.655	164	87	229
**9–1**	**243**	0.7397	0.728	938				0.7472	0.653	174	**89**	**216**
**9–2**		0.7370	0.732	925				0.7862	0.602	217		
**9–3**		0.7456	0.720	967				0.7604	0.642	187		
**average**		**0.7408**	**0.726**	**943**				**0.7646**	**0.632**	**193**		
10	228	0.7294	0.742	890				0.7502	0.655	178	86	196

**SPLIT3**			
**Subtraining set, n=165**	**Calibration set, n=167**	**Test set, n=61**	**SA_k_ distribution**

*[Subtraining-Calibration-Test] system Split3*												
limS	Nact	R^2^	s	F	R^2^	s	F	R^2^	s	F	W%	N_111_
0	797	0.8690	0.518	1084	0.8909	0.516	1353	0.5794	0.929	82	42	332
1	614	0.8742	0.508	1134	0.8946	0.513	1402	0.5995	0.896	89	50	309
2	402	0.8266	0.597	778	0.8331	0.614	826	0.6748	0.800	122	69	278
**3–1**	**324**	0.7963	0.647	637	0.7982	0.633	652	0.7176	0.729	150	**78**	**254**
**3–2**		0.7919	0.654	620	0.7937	0.639	635	0.6969	0.758	136		
**3–3**		0.7930	0.652	624	0.7944	0.641	637	0.7431	0.698	171		
**average**		**0.7937**	**0.651**	**627**	**0.7954**	**0.638**	**642**	**0.7192**	**0.728**	**152**		
4	264	0.7439	0.725	474	0.7462	0.703	485	0.6992	0.765	138	85	224
5	227	0.7127	0.768	404	0.7136	0.738	411	0.6900	0.774	133	86	195
6	198	0.6945	0.792	371	0.7013	0.756	388	0.6899	0.770	133	86	171
7	181	0.6790	0.812	345	0.6843	0.780	358	0.6995	0.758	137	85	154
8	159	0.6432	0.856	294	0.6493	0.815	306	0.7061	0.749	142	84	134
9	147	0.6219	0.881	268	0.6533	0.820	311	0.6934	0.775	134	84	123
10	140	0.5952	0.911	240	0.6269	0.849	277	0.6300	0.842	101	83	116

**Training set, n=332**	**Calibration set, n=0**	**Test set, n=61**	**SA_k_ distribution**

*[Training-Test] system Split3*												
limS	Nact	R^2^	s	F	R^2^	s	F	R^2^	s	F	W%	N_101_
0	797	0.8930	0.457	2756				0.5532	1.009	73	47	377
1	776	0.8932	0.457	2763				0.5529	0.996	73	46	356
2	540	0.8699	0.504	2209				0.5998	0.922	89	61	327
3	434	0.8349	0.568	1674				0.5908	0.896	88	72	311
4	388	0.8220	0.590	1528				0.6068	0.865	92	75	291
5	348	0.8030	0.620	1346				0.6650	0.796	117	78	272
**6–1**	**320**	0.7773	0.660	1152				0.7017	0.751	139	**82**	**261**
**6–2**		0.7942	0.634	1273				0.6967	0.761	136		
**6–3**		0.7834	0.651	1193				0.7171	0.735	150		
**average**		**0.7850**	**0.648**	**1206**				**0.7051**	**0.749**	**141**		
7	288	0.7598	0.685	1045				0.6807	0.778	126	84	241
8	271	0.7637	0.679	1067				0.6520	0.817	112	85	229
9	244	0.7318	0.724	901				0.6833	0.778	127	86	210
10	232	0.7288	0.728	887				0.6826	0.781	127	84	196

**Table 5.** Correlation weights for calculation with Equation 1 DCW(4). N(Subtr), N(calib), and N(Test) are numbers of a given SMILES attribute in the subtraining set, calibration set, and test set, respectively. The rare attributes are omitted.

**SMILES-Attributes (SA)**	**CW(SA) probe 1**	**CW(SA) probe 2**	**CW(SA) probe 3**	**N(Subtr)**	**N(Calib)**	**N(Test)**

**dC**						
!-01........	2.7522274	2.8704615	3.5346711	5	4	0
!-02........	1.2190257	2.1277910	1.8790680	10	9	3
!-03........	6.6784389	8.0311759	7.1271958	15	10	3
!-04........	1.4326102	1.6702225	1.9340790	17	22	8
!-05........	3.9671055	4.0344924	4.1729635	9	11	6
!-06........	5.8564637	5.8794012	6.4409754	8	11	7
!-07........	5.4970475	5.1611240	5.2308474	5	3	0
!-08........	9.1295923	9.5122328	9.0035813	4	3	1
!-21........	−1.6383248	1.8781962	0.0037831	4	0	0
!000........	3.6271821	4.6894405	3.7495506	6	7	1
!002........	1.5603260	1.7450611	1.4951171	4	4	1
!003........	−1.2514096	−1.3248590	−1.1256941	5	8	2
!004........	0.7359726	1.0643522	1.2450258	11	8	1
!005........	0.9702817	0.6240636	1.2144260	13	9	4
!006........	4.1543029	4.9338830	4.9975361	7	5	2
!007........	−3.7770327	−3.5039029	−3.0945823	4	3	3
!010........	0.5049355	−0.2636435	0.3157527	6	8	3
!012........	3.2511213	3.2471578	4.5049864	6	3	1

**^1^SA_k_**						

#...........	3.3706294	3.3739877	2.0643948	5	3	0
(...........	−1.6866726	−1.3666396	−1.5485382	708	780	260
/...........	−0.4913426	0.1880630	−1.0975733	17	24	4
1...........	−1.4970879	−0.8440743	−0.0771659	222	222	88
2...........	−0.1050677	−1.1891334	−1.1138329	130	132	48
3...........	−1.3433340	0.0456678	−0.1828115	60	60	20
4...........	3.4954870	3.1562107	3.5453447	20	18	8
5...........	2.8128037	3.3899959	1.6902086	10	10	4
=...........	−1.8660845	−2.1441609	−1.8865449	77	79	23
C...........	−0.0156855	0.0453525	0.2198595	765	736	290
Br..........	0.5327181	0.2779344	0.8454938	23	8	1
Cl..........	2.9838590	2.1906970	3.1890603	61	85	13
F...........	−0.4680666	−1.0425952	0.2836492	15	19	8
O=C.........	−2.8475657	−2.4376628	−2.9332073	33	21	13
O=..........	0.7369372	0.0037805	1.4086398	140	132	47
N...........	1.1227501	1.1982965	1.4193640	196	201	76
O...........	−1.2649109	−0.4408418	−0.1501499	138	143	45
S...........	2.3712714	2.6251760	2.5313565	13	12	7
[N+]........	1.9345689	1.6543771	3.0457447	26	31	12
[O−]........	5.9250900	5.6230564	6.9653600	26	32	12
[...........	−2.1531745	−2.8080919	−1.9966710	4	6	0
\...........	3.3565892	3.4338813	2.9027414	14	29	7
c...........	−0.0357264	0.0373181	0.0419142	653	679	247
n...........	−0.6564241	−0.1251570	−1.4184164	37	44	23
o...........	−1.0665085	−1.3777640	−0.2485470	16	12	7
s...........	−0.0527175	−0.9991370	−1.0040993	7	6	7

**^2^SA_k_**						

(...(.......	−0.0735964	−0.1751550	−0.4970432	18	28	4
/...(.......	−0.9972903	−1.5270762	−0.7479799	7	10	2
1...(.......	2.3733452	2.4975765	2.4084744	37	45	15
2...(.......	0.0608136	0.1227718	−0.1848792	14	15	6
2...1.......	5.7529509	6.6287931	7.4713129	5	6	2
3...(.......	−1.6283079	−0.2528134	−2.0499858	6	3	0
3...2.......	−1.5915226	−1.5268568	−1.9365673	4	6	3
=...(.......	1.8468333	2.3079839	2.4377851	14	17	3
=...1.......	2.5039102	−0.5608527	−0.0325572	7	5	1
=...2.......	−3.2847055	−2.5042239	−2.0658154	7	5	2
=...3.......	3.4389111	0.8278003	3.5625409	6	5	4
C...#.......	−0.1836222	−0.9385750	−0.4107562	6	3	0
C...(.......	−0.7573851	0.0280162	0.7466835	443	456	163
C.../.......	1.1245359	0.0042443	0.8873189	13	13	2
C...1.......	−0.4566262	0.0357389	−1.3911620	74	73	30
C...2.......	0.3115003	1.0911890	0.8401947	46	47	12
C...3.......	3.6534836	3.4678053	2.9866499	40	22	8
C...4.......	−0.7152591	−0.7967591	−1.2502575	17	13	5
C...5.......	3.6909807	4.4229382	4.3015822	10	7	6
C...=.......	−0.5319093	−0.5807285	−0.4569253	98	101	29
C...C.......	−0.4098212	−0.6663667	−0.4722713	244	211	113
Br..(.......	−1.2467411	−0.7804139	−0.9676602	24	7	0
Br..C.......	5.8039394	6.6601591	5.9721683	9	5	1
Cl..(.......	−0.2165917	−0.6443513	−0.7389015	68	104	11
Cl..C.......	6.8768666	7.6839570	7.4343341	17	18	5
F...(.......	0.2020867	−0.0538118	−0.1868874	24	22	12
O=C.(.......	0.7311485	−1.6257029	0.2188934	18	8	5
O=C.1.......	4.3778160	4.7809340	4.0011131	9	6	1
O=..(.......	−0.5612999	−1.5272716	−1.1454337	177	158	60
O=..1.......	−2.5019413	−3.3715192	−4.0028237	4	2	0
N...#.......	−3.8725309	−4.4992215	−3.7843832	4	2	0
N...(.......	0.0666245	0.7453778	−0.1289674	140	165	56
N.../.......	0.8133323	0.0606093	−0.1893841	9	12	2
N...1.......	1.8744868	1.0335496	1.5038557	23	17	10
N...2.......	1.4979132	1.4959901	1.4961647	6	9	3
N...=.......	−1.3157419	0.1537739	−0.3882898	12	16	5
N...C.......	1.4051238	0.9827410	1.0619180	63	70	24
N...O=......	6.1270291	7.3000058	4.8170313	39	34	13
N...N.......	3.1922498	3.5013150	4.1321688	14	8	8
O...(.......	−0.1195150	−0.1976562	−0.7838811	106	111	31
O...1.......	−0.7620380	−1.4388311	−1.9361803	19	13	5
O...2.......	−2.5618134	−3.2668394	−2.8747322	9	5	3
O...C.......	1.0444154	1.0339726	0.9105754	90	96	29
S...(.......	−0.7479741	0.4990928	−0.0132133	7	8	4
S...=.......	1.5009045	0.6752299	−0.2807681	5	7	2
S...C.......	0.2470117	−1.2535030	−0.5349209	6	1	4
[N+](.......	3.2516821	1.6524330	1.3748828	40	37	17
[O−](.......	−0.4532482	−0.8221590	−1.3547359	39	48	18
[O−][N+]....	0.2616708	0.6284804	−1.2536848	5	6	2
\...(.......	0.2506876	−0.8700648	−1.1268254	5	11	1
\...C.......	2.1710329	2.6262343	1.7619375	11	26	6
\...N.......	−3.1201815	−3.8706242	−3.0325970	4	12	3
c...(.......	0.3275817	−0.1910585	−0.5343311	183	238	94
c...1.......	0.5127781	0.1714980	0.8236519	196	204	75
c...2.......	0.1139969	1.4331593	2.2509927	129	122	38
c...3.......	1.5045372	1.4375414	0.1592669	41	50	15
c...4.......	0.9391582	0.2451376	0.7605772	9	10	10
c...C.......	−1.6459258	0.0580657	−0.4333240	15	19	1
c...Cl......	−1.9973422	−2.7517912	−3.6905785	5	7	3
c...N.......	1.0896408	0.1897391	0.7548234	26	19	12
c...O.......	2.4331156	1.2515997	0.9178503	22	18	6
c...c.......	−0.2252497	−0.6284915	−0.8624749	316	305	106
n...(.......	0.8765637	−0.9401023	0.2494319	11	8	6
n...1.......	1.6235455	1.2765370	1.8586068	16	15	10
n...2.......	2.0303385	3.9797145	3.5009942	6	11	8
n...3.......	4.1295873	3.5576218	4.1220666	4	6	3
n...c.......	2.3101715	1.5599987	1.7164852	25	40	17
o...(.......	−3.9990305	−2.9953875	−3.7613936	8	9	6
o...1.......	5.8875962	7.1857177	6.8437068	5	5	2
o...2.......	−0.6199000	−0.1044473	−0.2658548	7	6	5
o...c.......	5.5725605	4.2549084	3.0289124	8	3	1
s...1.......	0.9407152	1.9765325	2.0040980	6	6	7
s...c.......	−0.4960014	0.0005352	0.0003709	4	2	6

**^3^SA_k_**						

(...C...(...	3.9980286	3.5630634	2.4208800	95	102	42
(...Br..(...	0.0004754	0.5262619	−1.7321140	9	3	0
(...Cl..(...	0.6233843	−0.3777470	1.3569611	29	44	5
(...F...(...	1.9216525	2.4994253	1.1337512	11	9	5
(...O=..(...	1.2586684	0.9193678	0.2775945	71	68	28
(...N...(...	2.1549141	1.5500875	1.3741232	29	40	11
(...O...(...	1.3921586	0.5590769	0.3741692	33	34	8
(...[N+](...	2.2543551	4.4491588	4.0323477	15	8	5
(...[O−](...	−1.5354823	−1.4737180	−3.3779724	19	23	9
(...c...(...	−1.0930229	−0.9342219	0.4332127	12	18	0
/...C...(...	4.4970818	4.0048287	3.0000737	5	4	0
1...C...(...	4.2142525	2.8161885	3.1850279	16	16	5
1...O...(...	2.2528410	0.3797661	1.4952742	4	2	0
1...c...(...	0.9335129	0.5288083	0.8716499	18	35	12
2...C...(...	−2.2544539	−1.0919167	−2.6427067	10	13	4
2...c...(...	2.9972422	3.4417285	3.8400379	29	28	12
2...c...1...	1.9960734	0.5619955	−0.0932258	7	8	2
2...o...(...	1.0110528	1.4973281	1.0603210	4	4	4
3...C...(...	−2.2814415	−2.8156671	−1.9977117	7	6	1
3...C...2...	6.4980735	8.0020020	8.2529271	4	0	0
3...c...(...	−0.2171379	1.0578016	1.2483687	9	9	4
3...c...2...	5.2502023	4.2226554	4.7487134	8	7	2
4...C...(...	1.7464204	−0.6210801	−0.2842473	6	4	0
=...C...3...	7.0029958	7.5641676	6.7549811	8	4	0
=...C...1...	2.9999456	2.8474909	4.2545986	12	8	4
=...C...(...	1.5713076	0.9521538	0.5309943	18	18	2
=...C.../...	5.2495800	5.4994084	6.0049990	6	7	2
=...N.../...	5.8119346	5.5954944	6.3789368	7	8	2
C...(...C...	0.5513380	−0.2378199	−1.0864076	69	64	24
C...(...1...	−1.1216831	−3.3768328	−2.4951909	9	10	3
C...(...=...	6.2535976	5.1587497	4.4360925	9	11	3
C...(...(...	−0.3146880	−1.6918223	−1.2834724	11	22	3
C.../...(...	−3.0637430	−1.8160468	−2.9987839	5	4	1
C...1...C...	5.4954661	3.9189550	4.6140280	8	10	5
C...1...(...	1.3718529	1.3079333	1.4795097	8	13	1
C...1...=...	0.2476856	1.3169467	0.9333222	6	4	1
C...2...(...	−0.6449419	−0.8430901	−0.7370108	5	9	0
C...2...C...	5.9965379	5.9966533	6.0017257	8	6	1
C...2...=...	0.0028591	−0.8747150	−0.3764369	7	5	2
C...3...(...	6.5045056	6.2477966	5.7492216	5	3	0
C...3...=...	5.6231609	6.0002916	5.1293498	5	3	2
C...3...C...	−3.5000076	−2.9954231	−3.0028309	11	2	3
C...4...C...	−3.0021372	−4.5016046	−2.9968826	4	1	1
C...=...1...	0.4331526	2.6582434	1.9050556	7	5	1
C...=...(...	−2.4953051	−0.6283873	−0.7473193	8	11	1
C...=...C...	1.6093540	2.7975919	2.1897927	33	34	11
C...=...3...	0.2971809	1.5013043	−0.9639603	5	3	2
C...=...2...	1.6275637	3.7172258	1.7773288	7	4	0
C...C...3...	5.0746157	4.6119002	4.5908880	16	8	5
C...C...=...	−0.4018420	−1.0583619	−1.0977818	36	26	5
C...C...1...	1.8775937	0.9384077	1.3828674	31	27	17
C...C...2...	0.7079969	−0.1879112	0.6274962	21	19	3
C...C...(...	1.0123078	0.7924398	0.4047303	109	109	42
C...C...4...	−1.4024533	−0.7803070	−0.3715353	7	5	2
C...C...C...	−0.0428402	0.1882515	−0.1515135	77	58	59
C...Br..(...	1.6298040	2.5038504	1.0267176	4	1	0
C...Cl..(...	−1.4962815	−0.4993582	−1.2516847	4	4	1
C...N...1...	−0.2691219	−0.3795019	−1.0000894	8	8	1
C...N...(...	1.4422504	0.9731136	0.4339815	36	35	18
C...O...2...	5.1236472	4.0895698	3.5121038	5	3	1
C...O...(...	3.2515162	2.2843408	2.5780816	28	32	12
C...O...C...	4.3741698	4.3105041	3.0634685	8	10	4
C...O...1...	2.8733789	3.1267319	2.9673439	13	9	3
C...\...C...	−2.8461979	−3.8759584	−2.2789129	4	8	1
C...c...2...	6.0006860	4.5315715	5.2468516	4	5	0
C...c...1...	2.4356912	0.4351720	1.2472872	10	13	1
Br..(...C...	2.0615855	1.1437599	1.9040185	4	6	0
Br..C...(...	1.4981969	0.6256406	0.0009660	7	3	0
Cl..(...(...	−1.2075807	−0.6362162	−1.9255180	9	6	0
Cl..(...C...	−1.1526609	−0.2476848	−1.8147825	27	32	4
Cl..(...Cl..	0.5049208	3.2539441	0.6886852	4	7	0
Cl..C...C...	−0.0014586	0.0039516	−1.2464369	9	10	2
Cl..c...1...	−0.2533902	1.6295626	2.2512123	4	6	3
F...(...C...	1.6863754	1.5015167	0.5346605	5	8	2
F...(...(...	−1.7457403	−2.1139078	−1.1982770	6	8	4
O=C.1...C...	5.1279970	2.8669862	3.6914477	4	3	1
O=..(...C...	0.8107109	0.7780984	−0.4033174	92	68	31
O=..1...C...	−3.7510578	−4.1255552	−4.1222938	4	2	0
O=..N...(...	9.5435183	10.0636543	10.0315899	24	28	6
N...#...C...	−4.5000318	−4.5014930	−4.5004055	4	1	0
N...(...N...	1.1916803	0.9983579	1.6288201	12	10	2
N...(...1...	−0.1264931	−0.7538114	0.0454142	5	7	0
N...(...C...	3.5018836	2.2822748	3.0336305	55	62	30
N...(...O=..	−2.3138510	−2.3145054	−1.5031092	23	14	5
N...(...O=C.	−1.4990141	−0.8704228	−1.2494712	6	5	2
N...1...C...	2.6914072	2.5647275	2.7184638	12	13	4
N...2...C...	−0.4978517	−0.0000051	1.0021092	5	6	1
N...C...(...	−0.8104915	−0.4341890	−0.9684013	25	24	8
N...C...C...	−1.2520104	−0.7226801	−1.0008159	22	26	6
N...O=..(...	2.6715972	1.6272171	3.6550784	11	11	1
N...N...1...	0.0042593	1.4970138	1.1825822	5	3	3
N...N...O=..	4.2459870	3.7466101	3.2221499	10	6	5
N...N...(...	4.7536740	5.4055258	4.6294702	6	4	2
N...c...2...	−3.6269860	−2.8792235	−4.2532265	5	3	1
N...c...1...	−0.1899251	0.2338572	0.2627307	20	15	11
O...(...O=..	−0.6219316	1.4395827	1.2532594	19	17	5
O...(...C...	0.9395840	−0.4347300	0.4467587	52	40	17
O...(...(...	11.5040533	12.0049318	11.9989881	4	4	1
O...(...O=C.	4.9368982	4.9994000	4.7472261	7	2	0
O...C...1...	−0.4987416	0.4978181	0.9413176	4	4	3
O...C...C...	−2.6559832	−3.2412835	−3.2024047	35	37	10
O...C...(...	−0.1201364	−0.3106109	−1.2477221	27	31	10
O...c...1...	−2.7623334	−2.0920952	−2.1242060	14	8	3
O...c...2...	−1.4980614	−3.5286748	−3.6223778	7	6	0
S...C...C...	−0.0034408	1.5042803	0.7537994	4	0	2
[N+](...C...	9.2539066	9.4417355	7.5006239	6	4	1
[N+](...2...	5.4109375	4.6273127	3.2821267	6	3	4
[N+](...O=..	−0.3787790	0.3109436	0.1872916	4	7	2
[O−](...[N+]	−3.8743809	−1.4388021	−1.2453181	18	22	9
[O−](...O=..	−4.0585677	−2.6242009	−2.5577359	15	11	6
[O−][N+](...	−3.5045096	−1.4982980	−2.4892797	5	5	2
\...C...=...	−1.3136029	−1.8755430	−1.2854492	4	11	1
\...C...(...	−3.5018378	−4.4994516	−3.8096741	5	7	2
c...(...[O−]	3.9992170	4.0612326	4.4978479	4	10	3
c...(...c...	1.7523875	2.5921235	0.9359654	24	19	13
c...(...Br..	1.3392341	0.5340779	1.1889034	17	0	0
c...(...C...	1.0002010	0.2472155	−0.2478234	19	41	13
c...(...Cl..	1.1825597	2.5039425	0.9088978	15	23	7
c...(...O...	1.1553993	0.9107402	1.8401083	10	34	6
c...(...N...	−0.4647652	−0.5109250	0.5049629	13	41	11
c...(...1...	3.2546395	1.6826438	1.7521385	17	17	10
c...(...O=..	2.0008786	2.9044603	2.8172049	15	17	7
c...(...F...	2.5615727	2.2483847	2.9956341	6	0	2
c...1...O...	0.3157218	0.2460029	0.0026575	7	3	3
c...1...C...	−0.3426326	−0.4053179	−0.0587013	10	10	4
c...1...(...	4.1291472	3.7385617	4.5010102	15	17	6
c...1...c...	2.5270201	4.1254591	1.6392878	64	69	24
c...2...c...	3.1834674	3.5765233	2.5649848	46	41	10
c...2...O...	−2.1902681	−0.8169002	−1.5600959	6	5	0
c...2...C...	2.0599980	3.3166075	2.0508240	6	4	2
c...2...(...	−3.4837706	−2.7454921	−2.2497303	5	2	2
c...3...c...	0.5671953	0.3280375	2.5577465	14	15	6
c...C...C...	−0.4968006	−1.0630716	−0.7464899	4	6	0
c...N...(...	4.1212930	5.1280879	3.2342653	9	3	3
c...O...(...	7.8795041	8.7529500	8.5448576	5	2	0
c...O...C...	0.4969760	1.0602889	1.0035994	10	8	0
c...c...2...	−1.0046754	−0.7477295	−1.3148992	59	58	20
c...c...c...	−0.9189229	−1.1362229	−0.9886103	171	148	50
c...c...1...	0.9684404	1.0961687	1.0267859	111	101	36
c...c...3...	−1.4056269	−2.8661586	−1.7490548	18	24	6
c...c...4...	−1.2498300	0.6278968	0.4997288	5	5	4
c...c...(...	−0.5592802	−0.7452834	−0.2831183	87	110	45
c...n...1...	0.4037274	0.7545182	1.8635708	8	9	8
n...1...c...	1.1446162	1.1906216	0.1368885	11	8	8
n...c...c...	−4.4951810	−4.4955509	−4.2500468	5	11	1
n...c...(...	−1.7475062	−0.9098866	−0.0016730	10	13	13
o...(...c...	1.9983265	−0.3077248	1.1610603	5	5	5
o...1...(...	−0.8795536	−0.8151611	−1.4961309	4	3	2
s...1...(...	3.0007359	3.3126224	2.8719278	5	5	6

**Table 6.** Examples of compounds which contain promoters of increase/decrease of the logTD_50._

**Structure**	**CAS and SMILES**	**logTD_50_**
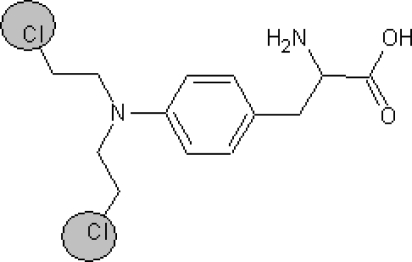	148-82-3 O=C(O)C(N)Cc1ccc(cc1)N(CCCl)CCCl	3.512
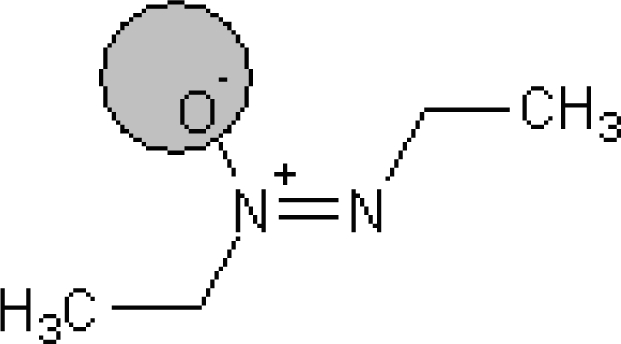	16301-26-1 [O−]\[N+](CC)=N\CC	3.667
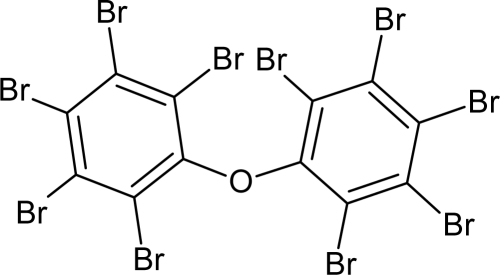	1163-19-5 Brc2c(Oc1c(Br)c(Br)c(Br)c(Br)c1Br)c(Br)c(Br)c(Br)c2Br	−0.542*
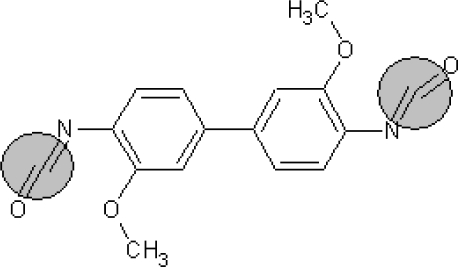	91-93-0 COc1cc(ccc1/N=C=O)c2ccc(\N=C=O)c(OC)c2	−0.740*

*) One can see that aromatic bonds are indicated in SMILES by ‘c’ (lower case), thus ‘=’ is indicator of local double bonds which are not a part of aromatic fragments.
